# Inhibition of emotions in healthy aging: age‐related differences in brain network connectivity

**DOI:** 10.1002/brb3.2052

**Published:** 2021-02-04

**Authors:** Ina S. Almdahl, Liva J. Martinussen, Ingrid Agartz, Kenneth Hugdahl, Maria S. Korsnes

**Affiliations:** ^1^ Department of Old Age Psychiatry Oslo University Hospital Oslo Norway; ^2^ Institute of Clinical Medicine Faculty of Medicine University of Oslo Oslo Norway; ^3^ Department of Psychology Faculty of Social Sciences University of Oslo Oslo Norway; ^4^ Department of Psychiatric Research Diakonhjemmet Hospital Oslo Norway; ^5^ Norwegian Centre for Mental Disorders Research (NORMENT) Institute of Clinical Medicine University of Oslo Oslo Norway; ^6^ Department of Clinical Neuroscience Centre for Psychiatric Research Karolinska Institutet Stockholm Sweden; ^7^ Department of Biological and Medical Psychology University of Bergen Bergen Norway; ^8^ Division of Psychiatry Haukeland University Hospital Bergen Norway; ^9^ Department of Radiology Haukeland University Hospital Bergen Norway

**Keywords:** aging, connectome, emotions, fMRI, inhibition

## Abstract

**Introduction:**

Successful inhibition of distracting emotions is important for preserving well‐being and daily functioning. There is conflicting evidence regarding the impact of healthy aging on emotional inhibition, and possible age‐related alterations in the neuronal underpinnings of emotional interference processing are unexplored.

**Methods:**

Thirty younger (mean age 26 years; 15 women) and 30 older (mean age 71 years; 13 women) healthy adults performed a face–word emotional Stroop task while undergoing functional magnetic resonance imaging of the brain. A resting‐state scan was acquired for calculating the amplitude of low‐frequency fluctuations as an estimate of vascular reactivity. Comparisons of brain activation during the task were assessed in a whole‐brain, voxel‐wise analysis, contrasting congruent, and incongruent conditions. The canonical regions of the frontoparietal, salience, dorsal attention, and default mode networks were used as seed regions for assessing functional connectivity within and between large‐scale brain networks. Task performance was evaluated using response accuracy and response time.

**Results:**

The older adults had longer response times and lower task accuracy than the younger adults, but the emotional interference effect was not significantly different between the groups. Whole‐brain analysis revealed no significant age‐related differences in brain activation patterns. Rescaling the data for estimated variability in vascular reactivity did not affect the results. In older adults, there was relatively stronger functional connectivity with the default mode network, the sensorimotor network, and the dorsal attention network for the frontoparietal and salience network seeds during the task. Conversely, younger adults had relatively stronger connections within and between the frontoparietal and salience networks.

**Conclusion:**

In this first fMRI study of emotional Stroop interference in older and younger adults, we found that the emotional interference effect was unchanged in healthy aging and replicated the finding from non‐emotional task studies that older adults have greater between‐network and less within‐network connectivity compared to younger adults.

## Graphical abstract

First fMRI study of face–word emotional Stroop interference in healthy aging. The interference effect did not change, and the basic pattern of brain activation was preserved with advancing adult age. The older adults had stronger between‐network connectivity and weaker within‐network connectivity of the major attention/control networks during the emotional interference task compared to younger adults.

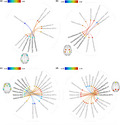

## 1 INTRODUCTION

Being able to ignore distracting emotional information and carry on with the task at hand is crucial in everyday life. We are often confronted with conflicting stimuli, and our brains promptly have to select what is relevant and what should be disregarded to avoid interference with goal achievement. According to the inhibitory deficit hypothesis, the capacity for such inhibitory cognitive control declines with increasing adult age (Hasher, 2015; Hasher & Zacks, 1988); however, meta‐analyses have called into question the notion of a general aging‐related inhibition deficit and pointed to more task‐specific differences (Rey‐Mermet & Gade, 2018; Verhaeghen, 2014). One of the most widely studied tasks of inhibition is the classic color–word Stroop interference task (Stroop, 1935), where correct color‐naming of a word is challenged by the predominant response to read the word. An interesting variant of the Stroop task involves including emotional material in the test as emotionally salient stimuli are particularly efficient distractors (Tipples & Sharma, 2000).

Many different versions of ‘emotional Stroop tasks’ have been studied and when reviewing the literature it is useful to classify the different task versions into two main groups: (a) tasks that include emotional stimuli, but without a direct semantic conflict or competing responses between task‐relevant and task‐irrelevant stimuli, such as color‐naming or counting of words that are either neutral or emotional (Ben‐Haim et al., 2016; Whalen et al., 2006) and (b) tasks where the interference occurs because of direct incongruity between task‐relevant and task‐irrelevant emotional stimuli, analogous to the classic cognitive Stroop test, such as the face–word emotional Stroop task (Etkin et al., 2006). For the first group of emotional Stroop tasks, the RT interference effect is reported to be small or even undetectable in healthy subjects (Algom et al., 2004; Dresler et al., 2012; Mama et al., 2013; Williams et al., 1996) and a meta‐analysis of neuroimaging studies found activation foci only in the precentral/postcentral gyrus for the interference contrast of these tasks in healthy adults (Song et al., 2017). Conversely, the second type of emotional Stroop tasks create a reliable interference effect with significantly prolonged RTs in the incongruent condition (Etkin et al., 2006; Preston & Stansfield, 2008) and is consistently related to a brain activation pattern that spans the dorsolateral prefrontal cortex, inferior frontal gyrus, dorsal anterior cingulate cortex, and medial prefrontal cortex (Song et al., 2017).

Some of these frontal areas implicated in emotional Stroop interference also exhibit disproportionate gray and white matter volume loss in studies on healthy aging (Fjell et al., 2009, 2013; Raz et al., 2010). Behaviorally, the interactions between age and emotion are highly complex. Cross‐sectional studies have found evidence of improvements in emotional well‐being with age (Carstensen et al., 2011), and in certain cognitive tasks an aging‐related “positivity effect” has been observed, wherein older adults display facilitated attention and memory for positive relative to negative information (Barber et al., 2020; Leclerc & Kensinger, 2008; Mather & Carstensen, 2005; Reed & Carstensen, 2012; Sasse et al., 2014). This is in contrast to the fact that late‐life depression is a major public health challenge (Horackova et al., 2019). Against this backdrop, it appears particularly pertinent to assess whether interference in the emotional Stroop task changes with normal aging, and if so, to reveal the underlying neural structures that exhibit age‐related alterations in activity during the performance of the task. There are studies that have compared emotional Stroop performance in older and younger groups, but the majority of these have used emotional Stroop tasks without a direct semantic or response selection conflict in the emotional content (Ashley & Swick, 2009; De Raedt & Van Der Speeten, 2008; Jain & Labouvie‐Vief, 2010; Kappes & Bermeitinger, 2016; Lamonica et al., 2010; MacKay et al., 2015). Only a few studies have compared older and younger adults using emotional Stroop tasks that involve direct semantic conflict; some reported increased emotional interference in the older group (Agusti et al., 2017; Wurm et al., 2004), while others have found no (Berger et al., 2019) or reduced differences in RT interference with age (Jiang et al., 2007). These inconsistencies prove that further studies are needed to determine the impact of aging on emotional interference processing. Furthermore, none of these previous studies included neuroimaging. The current study aims to fill this gap in the literature by using functional magnetic resonance imaging (fMRI) to assess brain activation during an emotional face–word Stroop task in older and younger adults.

When using fMRI to assess neuronal activation patterns in older and younger groups, there is one obvious caveat: the blood–oxygen‐level‐dependent (BOLD) signal that forms the basis for fMRI is not a direct measure of neuronal activity, as it also depends on the vasculature's ability to respond to vasoactive stimuli such as increased neuronal activity. Vascular reactivity varies between individuals and between brain regions, and this variability may increase with age (Lu et al., 2011). To mitigate the effects of this variability, one can scale the BOLD signal by an estimate of vascular reactivity. Vascular reactivity has been estimated by measuring BOLD signal change in response to controlled CO_2_ inhalation or breath‐holding. These approaches have their disadvantages: CO_2_ inhalation requires extra equipment during scanning and is not tolerated by all subjects. Breath‐holding relies on compliance (21% of older adults in one study could not perform breath‐holding correctly (Jahanian et al., 2017)), is dependent on lung capacity (which can change with age), and is prone to motion artifacts. Both methods may themselves evoke some neuronal activation. Even without any intervention, there are spontaneous fluctuations in arterial CO_2_ levels in the normal resting state. This is reflected in the amplitude of the low‐frequency fluctuations in resting‐state fMRI (RSFA), which has been shown to be highly correlated with end‐tidal CO_2_ measurements (Golestani et al., 2015; Wise et al., 2004). Calculating the variations in RSFA and using it as an indicator of vascular reactivity has been reported to compare favorably with CO_2_ inhalation and breath‐holding studies (Kannurpatti & Biswal, 2008; Kannurpatti et al., 2011; Liu et al., 2017; Tsvetanov et al., 2015). We therefore present BOLD activation data with and without scaling for RSFA.

Beyond changes in the activity of individual cortical areas during a task, age‐related alterations can also impact interactions between different brain regions and the role of overarching brain networks when performing the task. This can be probed by assessing fMRI functional connectivity, that is, the temporal correlation between the BOLD signal time series in spatially separate brain areas. The most consistent observation in previous functional network connectivity studies of aging with other tasks is that older adults seem to have lower within‐ and greater between‐network connectivity in the canonical cognitive brain networks compared to younger individuals (Damoiseaux, 2017; Dorum et al., 2016; Grady et al., 2016; Spreng et al., 2016). This supports the theory that aging of the brain is associated with neural dedifferentiation (i.e., reduced selectivity, where diverse cognitive processes become increasingly reliant on the same neural substrates with advancing age; Koen & Rugg, 2019). This pattern has been observed in a variety of cognitive tasks, but there is a dearth of studies assessing age‐related network connectivity changes during tasks involving emotional content. In a meta‐analysis of fMRI‐task studies on mainly young adults, the clusters activated during emotional interference processing predominantly mapped onto the frontoparietal network (FPN), the dorsal attention network (DAN), and the ventral attention/salience network (Chen et al., 2018). These three large‐scale brain networks are known to be involved in cognitive (non‐emotional) executive control, but some of their constituent regions have rich connections with the limbic regions of the brain. The FPN and salience networks have also been functionally associated with elements of emotion regulation (Lamke et al., 2014; Pan et al., 2018; Toller et al., 2018; Viviani, 2013). In particular, the salience network, which includes regions in the anterior cingulate cortex, anterior insula, and prefrontal cortex, has been implicated in modulating the activity of cognitive networks in response to emotional stimuli. The triple network model proposes that the salience network serves to identify salient stimuli and subsequently modify the balance between activity in the externally directed FPN and the internally directed default mode network (DMN), and that a wide range of psychopathologies can stem from aberrations in this system (Menon, 2011). By analyzing resting‐state fMRI, Nashiro et al. found disruptions in the functional connectivity of cognitive, motor, and visual networks with advancing age, but no effect of age on emotional networks (Nashiro et al., 2017). This raises the question of whether the age‐related network dedifferentiation previously described during the processing of cognitive tasks (a shift toward greater cross‐talk between networks and weaker within‐network integrity) will also occur for an executive control task that involves emotional content. The present study aims to answer this by assessing connectivity within and between the hubs of the canonical large‐scale brain networks. Based on prior studies, the regions of the three networks identified in the aforementioned meta‐analysis of emotional interference, (Chen et al., 2018) the salience network, FPN, and DAN as well as the DMN, were selected a priori as seed regions of interest (ROIs). As emotion processing in some aspects appears to be more preserved with aging than other cognitive modalities, determining whether the age‐related pattern of functional network restructuring is distinct for emotional tasks will contribute to our understanding of brain aging.

## 2 MATERIAL AND METHODS

### 2.1 Participants and screening assessments

The study sample consisted of 30 younger adults (aged 18–37 at the year of inclusion, mean age: 25.9 years, standard deviation [*SD*]: 5.3, 15 women) and 30 older adults (age range: 60–88 years, mean age: 70.8, *SD*: 7.4, 13 women) recruited through advertisements for healthy volunteers on posters distributed in and around the city of Oslo, Norway. Inclusion criteria, other than age, were as follows: no personal concerns regarding memory or other cognitive functions, no current or previous depressive episode or other significant psychiatric disease, fluency in the Norwegian language, and normal or corrected‐to‐normal visual acuity. Exclusion criteria were any medical disorders or medication/substance use deemed capable of potentially influencing cognitive functions based on clinical judgment, contraindications for MRI, or inability to complete the emotional Stroop fMRI‐task with sufficient image quality and with less than ± 2.5 mm translational or ± 2.5° rotational movement during the task. The participants were interviewed thoroughly about current and previous medical history. They completed a battery of neuropsychological tests: the revised Norwegian version of the Mini Mental Status Examination (MMSE‐NR3; Folstein et al., 1975); the clock‐drawing test; the Norwegian version of the Montreal Cognitive Assessment (MoCA; Nasreddine et al., 2005); the digit span forward and backward test from the Norwegian version of the Wechsler Adult Intelligence Scale, third edition (Wechsler, 2003); the short form of the Norwegian version of the California Verbal Learning Test, second edition (CVLT‐II; Delis et al.., 2000); the Trail‐Making‐Test A&B (Reitan, 1958); the Rey Complex Figure test (RCFT; Meyers & Meyers, 1995); the letter fluency test; and the classic color–word Stroop from Delis‐Kaplan Executive Function System (Delis et al., 2001). If needed, corroborative information was collected from medical records or interview with an informant. A modified version of the Framingham Heart Study 10‐year general cardiovascular disease risk score (D'Agostino et al., 2008) was calculated for each participant based on sex, systolic blood pressure, treatment for hypertension, smoking status, incidence of diabetes, and body mass index, with age set at 50 years for all participants. All participants provided written informed consent before partaking in any assessments. The study was conducted in accordance with the ethical principles stated in the Declaration of Helsinki. This study is a part of the DEPDEM‐project at Oslo University Hospital and was approved by the South East Regional Committee for Medical and Health Research Ethics in Norway [Reference No. 2016/1938].

### 2.2 Emotional Stroop task paradigm

**FIGURE 1:**
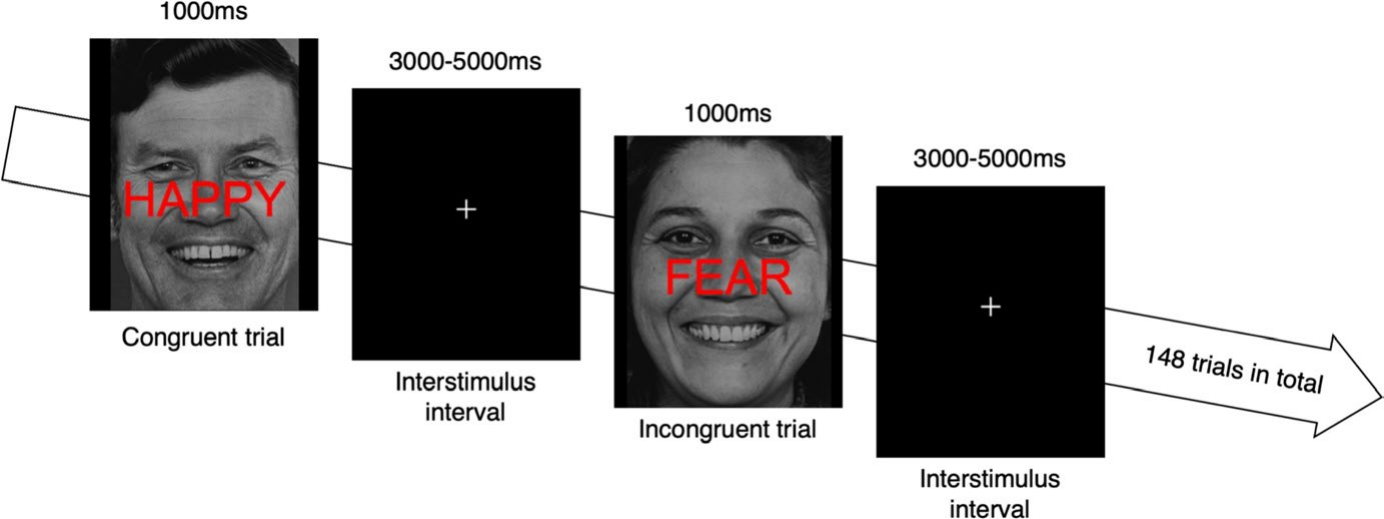
Illustration of the face/word emotional Stroop task (eStroop)

We used the emotional Stroop protocol developed by Etkin et al. (2006; abbreviated as eStroop). In this paradigm, the participants view photographs of faces expressing happiness or fear (from the picture set of Ekman and Friesen (1976)) with the word happy or fear written in capital red letters across the face. In our study, the words were written in Norwegian (“GLEDE”=happy, “FRYKT”=fear). The presented word in each trial was either congruent or incongruent with the emotional expression of the face. The participants were asked to identify the facial expression while ignoring the word. For incongruent trials, there was a direct semantic conflict between the task‐relevant facial emotion and the task‐irrelevant emotional word. For congruent trials, the word and the face expressed the same emotion. There were 148 face–word trials; half of these were incongruent, and half were congruent (Figure 1).

The trials were presented in pseudorandom order without direct repetition of the same face or the same face–word combinations. The stimuli were presented using E‐Prime®2.0 (Psychology Software Tools, Sharpsburg, PA) and displayed on an MR‐compatible LCD screen (NordicNeuroLab, Bergen, Norway) located behind the scanner bore. Each stimulus (face–word trial) was presented for 1 s, followed by a varying interstimulus interval of 3–5 s during which a central fixation cross was displayed. The total duration of trials and interstimulus intervals was 12 min and 27 s. The participants were instructed to answer as quickly and as accurately as possible. Responses were registered using hand‐held response buttons (ResponseGrip®, NordicNeuroLab, Bergen, Norway). Participants were pseudorandomized (based on odd or even numbers assigned consecutively at the time of inclusion) to instructions of pressing the right index finger for faces expressing happiness and the left index finger for faces expressing fear, or vice versa. The participants completed a short test version (10 trials) of the eStroop task before entering the MR scanner. Instructions were repeated before sequence initiation. A trigger pulse from the scanner synchronized the beginning of the task with the first fMRI volume. Trials with registered RT less than 300 ms were removed (three trials in the total set of 8,880 trials). Response accuracy and RTs (with and without exclusion of error trials) were exported for further analysis.

### 2.3 MRI acquisition

The participants were scanned using a GE Discovery MR750 3.0T scanner with a 32‐channel head coil. The participants wore earphones, and soft padding was placed between the earphones and the head coil to minimize head movements. A mirror was mounted on the head coil to allow the participants to view the LCD monitor located behind the scanner, ensuring that participants were able to see both the top and bottom of the screen in the resting position. Efforts were made to make the participants as comfortable as possible in the scanner before starting the scan. Participants were instructed to refrain, as far as possible, from moving during the scan. Before the task, a resting‐state gradient echo planar imaging (EPI) sequence was acquired with 200 volumes, 43 axial slices, TR 2,250 ms, TE 30 ms, flip angle 79°, field of view 256 × 256, matrix 96 × 96, voxel size 2.67 × 2.67 × 3, and total time 7.5 min. Participants were asked to keep their eyes open throughout the resting scan. The EPI scan during the eStroop task was acquired with 380 volumes, 38 axial slices, TR 2,000 ms, TE 25 ms, flip angle 90°, field of view 220 × 220 mm, matrix 64 × 64, voxel size 3.4375 × 3.4375 × 3.5, and total time 12.67 min. For both scans, the slices were acquired in interleaved order with a 0.5 mm inter‐slice gap. To obtain steady‐state magnetization, the EPI sequences were preceded by five volumes (dummy cycles) that were automatically discarded. The last volume of the eStroop scan (after completion of the task) was also discarded because of significant end of scan movement by two participants. Structural data were acquired using a sagittal T1‐weighted BRAVO sequence with 188 slices, TR 8.16 ms, TE 3.18 ms, flip angle 12°, field of view 256 × 256 mm, and voxel size 1 × 1 × 1 mm.

### 2.4 Analysis of task‐evoked activations

fMRI data for assessment of task‐related activations were pre‐processed and analyzed using the SPM12 software package (The Wellcome Centre for Human Neuroimaging, London, UK) in MATLAB® R2015b (MathWorks®, Natick, MA). The raw DICOM images were first converted to the NIfTI‐format before single‐subject pre‐processing. Slice‐timing correction for interleaved acquisition was performed before the time series were realigned with a six‐parameter rigid‐body spatial transformation with the aim of removing movement artifacts and unwarped to correct for movement‐related geometric distortions. The functional images were subsequently co‐registered with the structural scan and normalized to an MNI‐space template. Finally, the functional images were smoothed with a Gaussian kernel of 8 mm^3^ full width at half maximum (FWHM). To facilitate comparison with other studies, default SPM12 settings were used unless otherwise stated. A within‐subject general linear model (GLM) design matrix was specified with the congruent, incongruent, and error trials modeled separately as events. The error trials comprised both errors of commission (wrong responses) and errors of omission (no responses). In addition, the six movement regressors from the realignment procedure were included as covariates of no interest. Each event was convolved with a canonical hemodynamic response function. A temporal high‐pass filter of 1/128 Hz was applied to remove slow signal drifts. Temporal autocorrelation modeling was performed using FAST, a method that was recently shown to be superior to SPM’s standard pre‐whitening method (Olszowy et al., 2019). Voxel‐wise GLM parameters for each participant were estimated using restricted maximum likelihood, and then *t*‐statistics were computed for the contrast of interest, that is, incongruent versus congruent trials. The resulting contrast images were then assessed using a one‐sample *t* test for the whole sample and a two‐sample *t* test comparing younger and older adults. Voxel‐wise inference was performed using SnPM (version 13.1.08, http://nisox.org/Software/SnPM13/) with 10 000 permutations and a family‐wise error (FWE)‐corrected *p*‐value threshold <0 .05. Regions of significant activations were labeled by mapping the MNI coordinates of the peak voxels on to Brodmann areas using the tracing tool in the Yale BioImage Suite Package (Lacadie et al., 2008) and onto anatomical regions defined in the newest version of the Automated Anatomical Labelling Atlas (AAL3; Rolls et al., 2020) implemented in SPM12. The bspmview (Spun, 2016) SPM‐toolbox was used to generate surface renderings of the activations.

### 2.5 Scaling for resting‐state amplitude fluctuations

The resting‐state scans were pre‐processed using the same steps as the task fMRI scans. In addition, the component‐based noise correction method implemented in the CONN toolbox (Whitfield‐Gabrieli & Nieto‐Castanon, 2012) was employed, with five components each from the cerebrospinal fluid (CSF) and white matter (WM), the six regressors from the realignment and their first‐order temporal derivatives, as well as the effect of rest with its first‐order derivative as confounds. The fMRI signal was linear detrended, despiked, and band‐pass filtered (0.01–0.08 Hz). These processing steps are similar to those used in a study by Tsvetanov et al. (Tsvetanov et al., 2015) for a cohort of 335 adults, in which they demonstrated it to be a useful way of estimating RSFA compared with control measures of cardiovascular function and resting‐state magnetoencephalography. The frequency range chosen for the band‐pass filter has also been shown to be well correlated with the end‐tidal CO_2_ time course (Liu et al., 2017). RSFA was calculated as the standard deviation of the confound‐corrected resting‐state time series. Scaling was performed by dividing the parameter estimates for the task‐evoked responses (separately for congruent and incongruent trials) by the RSFA value for the same voxel.

### 2.6 Analysis of functional network connectivity during the eStroop task

Pre‐processing before ROI‐to‐ROI connectivity analyses was performed using the CONN toolbox—the functional images were realigned, unwarped, and slice‐time corrected. CONN’s ART‐based identification of outlier functional scans for scrubbing was used, with a scan‐to‐scan motion threshold of 0.9 mm and a global signal z‐value threshold of 5 (the default settings in CONN). Both functional and structural scans were directly segmented and normalized to the MNI‐space. Total gray and white matter volumes were estimated from the number of voxels included in the gray and white matter masks produced by the segmentation. Finally, the functional images were smoothed with an 8 mm FWHM kernel. The target resolution was 2 mm for the functional images and 1 mm for the structural images. Similar to the pre‐processing of the resting‐state scans, component‐based noise correction was executed with the following confounds: five components each from CSF and WM and 20 scrubbing components without derivatives, the six realignment parameters, and the baseline and task congruent, incongruent, and error conditions with their first‐order derivatives. The images were linear detrended and high‐pass filtered (>0.008 Hz). Functional connectivity analysis was performed as a weighted GLM with bivariate correlations and hemodynamic response function weighting for the task conditions. All 32 canonical network ROIs included in the CONN functional network atlas were selected as target ROIs: four DMN, three sensorimotor, four visual, seven salience, four DAN, four FPN, four language, and two cerebellar network nodes. These ROIs have been defined based on CONN’s ICA analyses of the Human Connectome Project dataset (497 subjects). As a previous meta‐analysis had found that clusters activated during emotional interference processing predominantly map onto the frontoparietal, the ventral attention/salience and the dorsal attention networks (Chen et al., 2018), the regions of these three networks were selected as seed regions in addition to the DMN ROIs. Connectivity within and between the network ROIs during the incongruent trials was compared between the older and younger groups using a two‐sample *t* test. The significance threshold was set at the false discovery rate (FDR)‐corrected *p*‐value < 0.05 at analysis‐level (correcting for both multiple seed and multiple target ROIs).

### 2.7 Statistical analyses

Demographic data and cognitive scores were analyzed using the *χ* ^2^‐test for categorical variables and the Mann–Whitney *U* test for continuous variables. Nonparametric tests were used because these scores are generally not normally distributed. For behavioral performance in the eStroop task, overall accuracy and RTs were compared between younger and older adults using the Mann–Whitney *U* test. To compare the time for correct responses in the congruent and incongruent trials between groups, a 2x2 mixed analysis of variance (ANOVA) was performed following log‐transformation of the raw RTs to approximate a normal distribution. These analyses were performed using SPSS version 25 (SPSS Statistics, IBM). For accuracy, the distributions were also skewed after log‐transformation, and the Brunner & Langer nonparametric model was applied using the nparLD package (Noguchi et al., 2012) in R version 3.6.3 (R‐Core‐Team, 2020).

## 3 RESULTS

### 3.1 Demographics, neuropsychological scores, and eStroop task performance

The demographic data and clinical scores for the groups are summarized in Table 1. The two groups had comparable scores on the Montgomery–Asberg Depression Rating Scale (MADRS; Montgomery & Asberg, 1979), while the younger adults on average scored slightly higher on the Geriatric Anxiety Inventory (GAI; Pachana et al., 2007). Table 1 also contains clinical data regarding blood pressure, body mass index, medication use, and sleep duration and quality (based on the Pittsburgh Sleep Quality Index [PSQI], all of which have been suggested to potentially influence the BOLD signal (Specht, 2019)). Unsurprisingly, the older adults had higher average systolic blood pressure and regularly used more medications than the young adults. The main classes of medications used were contraceptives in the young group (eight of the 30 participants), proton pump inhibitors (one in the young group and five in the older group), antihypertensive medications (seven older adults), and lipid‐lowering medications (five older adults). There were no significant group differences in terms of the time of the year or the time of the day the fMRI scans were acquired.

**TABLE 1:** Demographic data and clinical scores

	Younger adults	Older adults	*χ* ^2^/Mann–Whitney *U*	*p‐value*
*N*	30	30		
Age Age range	25.9 (5.3) <18–37>	70.8 (7.4) <60–88>		
Men/Women (*N*)	15/15	17/13	0.268	0.796
Educational level (years)	15.2 (1.7)	15.9 (2.7)	332.0	0.074
MADRS Depression score	0.6 (0.9)	0.4 (1.0)	390.5	0.305
GAI Anxiety score GAI Range	1.0 (1.5) <0–5>	0.2 (0.6) <0–3>	317.5	0.014*
Systolic blood pressure	118.8 (13.6)	138.2 (21.0)	201.0	<0.001*
Diastolic blood pressure	72.6 (8.9)	78.1 (12.2)	314.0	0.044
Body mass index	23.8 (3.5)	25.1 (3.1)	330.0	0.077
Medications regularly used (*N*)	0.3 (0.5)	1.4 (1.7)	295.5	0.010*
Medications range	<0–2>	<0–5>		
Sleep duration (PSQI−4, hours)	7.3 (0.8)	6.9 (0.9)	325.0	0.062
Sleep score (Global PSQI)	3.3 (1.9)	4.3 (3.5)	389.5	0.366

Note

Values are reported as mean (standard deviation) unless otherwise stated. Significant group differences after false discovery rate correction for multiple comparisons (*p* < 0.05) are asterisked.

The neuropsychological test results in the two groups are listed in Table 2. Briefly, in terms of the general cognitive screening tests (MMSE and MoCA), there were no significant differences between the groups. As expected, the younger group performed better in several of the individual cognitive tests, especially tests where scores depend on processing speed (the trail‐making test and the classic Stroop test) and memory (delayed recall of words and figure details), while there were no age‐related differences in verbal fluency or figure copy. Based on the neuropsychological test results and all other available assessment information, none of the participants fulfilled the NIA/AA criteria for mild cognitive impairment or dementia (Albert et al., 2011).

**TABLE 2:** Neuropsychological scores

	Younger adults	Older adults	Mann–Whitney *U*	*p‐value*
MMSE (max 30)	29.5 (0.6)	29.1 (0.9)	320.0	0.039
MoCA (max 30)	27.9 (1.7)	26.9 (2.0)	309.0	0.033
Clock‐drawing test (max 5)	4.9 (0.5)	4.7 (0.7)	379.0	0.145
Digit span (max 28)	16.2 (3.1)	14.6 (2.5)	325.0	0.062
Verbal list learning (short CVLT, max 36)	31.1 (2.9)	28.0 (3.2)	217.5	<0.001*
Verbal list delayed recall (max 9)	8.4 (0.8)	7.0 (1.2)	160.0	<0.001*
Complex figure (RCFT) copy (max 36)	33.2 (2.3)	32.0 (3.1)	343.5	0.109
Complex figure delayed recall (max 36)	22.1 (4.7)	13.8 (5.6)	125.5	<0.001*
Trail‐making test A (sec.)	21.7 (7.7)	33.8 (14.8)	165.5	<0.001*
Trail‐making test B (sec.)	58.9 (27.9)	83.7 (29.9)	207.0	<0.001*
Letter fluency	47.5 (10.1)	44.1 (11.8)	374.0	0.264
Classic Stroop 1 color‐naming (sec.)	28.5 (4.3)	33.6 (6.1)	206.0	<0.001*
Classic Stroop 2 word‐reading (sec.)	20.9 (3.1)	23.1 (4.2)	316.5	0.047
Classic Stroop 3 interference (sec.)	47.4 (10.3)	63.8 (15.3)	133.0	<0.001*
Classic Stroop 4 switching (sec.)	51.5 (9.1)	70.1 (22.0)	157.5	<0.001*
Classic Stroop 3 in % of Stroop 1	166% (22)	193% (53)	241.5	0.002*

Note

Values are reported as mean (standard deviation) unless otherwise stated. Significant group differences after false discovery rate correction for multiple comparisons (*p* < 0.05) are asterisked.

**FIGURE 2:**
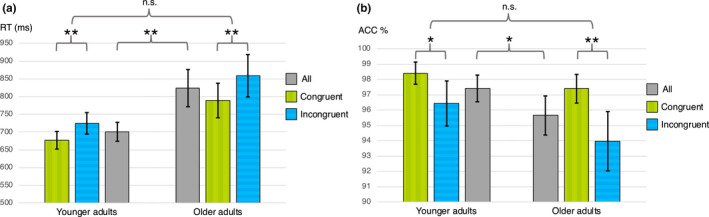
Performance in the eStroop task. The figure displays mean response times (RTs) for all correct trials (a) and mean accuracy (ACC) (b) in the two age groups separately for congruent, incongruent, and all trials, as well as the comparisons between the means within and between groups (*p < 0.05, **p < 0.001, n.s. = non‐significant). The error bars represent the 95%‐confidence intervals for the means. The *x*‐axes are cropped at 500 ms in (a) and 90% in (b) to improve visibility

The results of the eStroop task are presented visually in Figure 2 and numerically in Table 3. The correlation between mean ACC and RT was the same in the older adults (Spearman's ρ: −0.45, *p* = 0.013) as in the younger adults (Spearman's ρ: −0.46, *p* = 0.011). The absence of a significant difference in the RT interference effect between groups persisted even after including sex and educational level as covariates.

**TABLE 3:** Performance in the eStroop task

	Younger adults	Older adults	Mann–Whitney *U*	*Cohen's d*	*p‐value*
RT of correct trials (ms)	700.7 (72.8)	823.7 (141.7)	164.0	1.303	<0.001*
ACC (% correct responses)	97.4 (2.3)	95.7 (3.4)	278.0	0.695	0.010*
Errors of omission (*n* trials)	1.1 (1.8)	2.0 (1.5)	256.0	0.797	0.003*
Errors of commission (*n* trials)	2.8 (3.1)	4.4 (4.4)	309.0	0.559	0.035
Relative RT interference effect (%)	7.2 (3.9)	8.7 (5.4)	369.0	0.313	0.236
ACC interference effect (%)	2.0 (4.1)	3.4 (4.6)	339.5	0.432	0.103

Note

Values are reported as mean (standard deviation). Significant group differences after false discovery rate correction for multiple comparisons (*p* < 0.05) are asterisked. The error rate is separated in terms of trials without a response (omissions) and trials with an incorrect response. The relative RT interference effect is the difference in mean RTs for correct incongruent and correct congruent trials, in percentage of mean RT of correct congruent trials. The interference effect in ACC is the difference in the percentage of correct responses between incongruent and congruent trials.

Abbreviations: RT, response time; ACC, accuracy.

The mixed ANOVA of *congruency (congruent/incongruent) x group (younger/older)* for RT on correct trials revealed significant effects of congruency (*F(df 1) = *178.1, *p* < 0.001) and age group (*F(df 1) *= 20.7, *p* < 0.001), but there was no significant interaction between the two (*F (df 1) *= 1.5, *p* = 0.222). The same was true for ACC—there were significant effects of congruency (*F(df 1) *= 17.9, *p* < 0.001) and age group (*F(df 1) *= 8.5, *p* = 0.003), but no significant interaction (*F(df 1) *= 1.0, *p* = 0.322). To control for the effect of differences in processing speed between the groups, the time for completion of the trail‐making test A was entered as a covariate in the analysis of RT; however, this did not change the results. The RT interference effect was greater when the faces had a fearful expression (9.2% increase in RTs for incongruent trials, *SD* = 5.1%) than when faces expressed happiness (6.8%, *SD* = 6.3%), (*F(df 1) *= 8.809, *p* = 0.004), but there was no *valence x age group* interaction (*F(df 1) *= 1.256, *p* = 0.267). Regarding ACC interference, there was no statistically significant difference in terms of the emotional valence of the facial expression. Neither RT nor ACC was different for incongruent trials dependent on the sequence of trials (incongruent trials preceded by a congruent trial versus an incongruent trial).

Average inter‐scan movement during the eStroop task fMRI was higher in the older group (0.13 mm, *SD* = 0.04, CI = [0.12–0.15]) than in the younger group (0.08 mm, *SD* = 0.03, CI = [0.06–0.09]) (Mann–Whitney *U* = 103, *p* < 0.001). The number of scans removed during scrubbing before the functional connectivity analyses was also higher in the older group (142 censored scans of the total 11,370 scans, average 4.7 per participant, min 0, max 20) than in the younger group (71 censored scans, average 2.4 per participant, min 0, max 18) (Mann–Whitney *U* = 308, *p* = 0.025). Average inter‐scan movement was negatively correlated with average ACC and positively correlated with average RT of all trials, but only in the young group (motion x ACC Spearman's ρ: −0.46, *p* = 0.010; motion x RT; Spearman's ρ: 0.47, *p* = 0.010) and not in the older group (motion x ACC Spearman's ρ: −0.03, p = 0.868; motion x RT Spearman's ρ: −0.15, *p* = 0.432).

### 3.2 Voxel‐wise analysis of activations during the eStroop task

**FIGURE 3:**
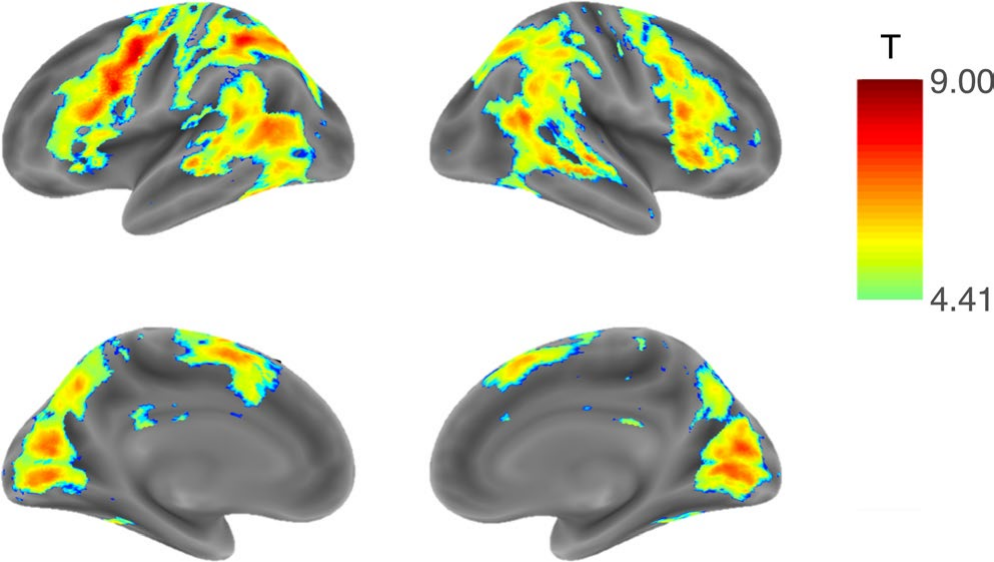
Results of the voxel‐wise one‐sample *t* test for the incongruent > congruent contrast across all 60 participants. Nonparametric correction for multiple comparisons using randomization with 10,000 permutations, FWE‐corrected *p* < 0.05, t‐threshold 4.41

The first overall analysis compared BOLD signal differences corresponding to the contrast between incongruent and congruent trials for the complete sample. Figure 3 shows the results of the whole‐brain voxel‐wise analysis for the entire sample of 60 participants. Activation for the incongruent > congruent contrast is seen mainly in the inferior frontal gyrus, precentral gyrus/supplementary motor area, middle temporal cortex, insula, inferior temporal/fusiform gyrus, and primary visual and inferior parietal cortices. There were no areas of significant deactivation for this contrast.

For the comparison between younger and older participants, no voxels revealed significant differences in activation at FWE‐corrected *p* < 0.05 (*u *≥ 4.6) at the whole‐brain level for the contrast between incongruent and congruent trials. For a comprehensive analysis, an exploratory comparison was also performed with uncorrected nonparametric *p* < 0.001 (thresholds to the result of permutation tests applied at each voxel), revealing small bilateral clusters of seemingly increased activation in the older group, primarily in the inferior frontal gyrus (Supporting Information, Table S1 and Figure S1).

### 3.3 Rescaling for resting‐state amplitude fluctuations

Controlling for variation in resting‐state amplitude fluctuations resulted in higher *t*‐statistics, but no voxels surpassed the FWE‐corrected *p* < 0.05 threshold on the whole‐brain level for the group comparison (Supporting Information, Table S2).

### 3.4 Functional network connectivity

**FIGURE 4:**
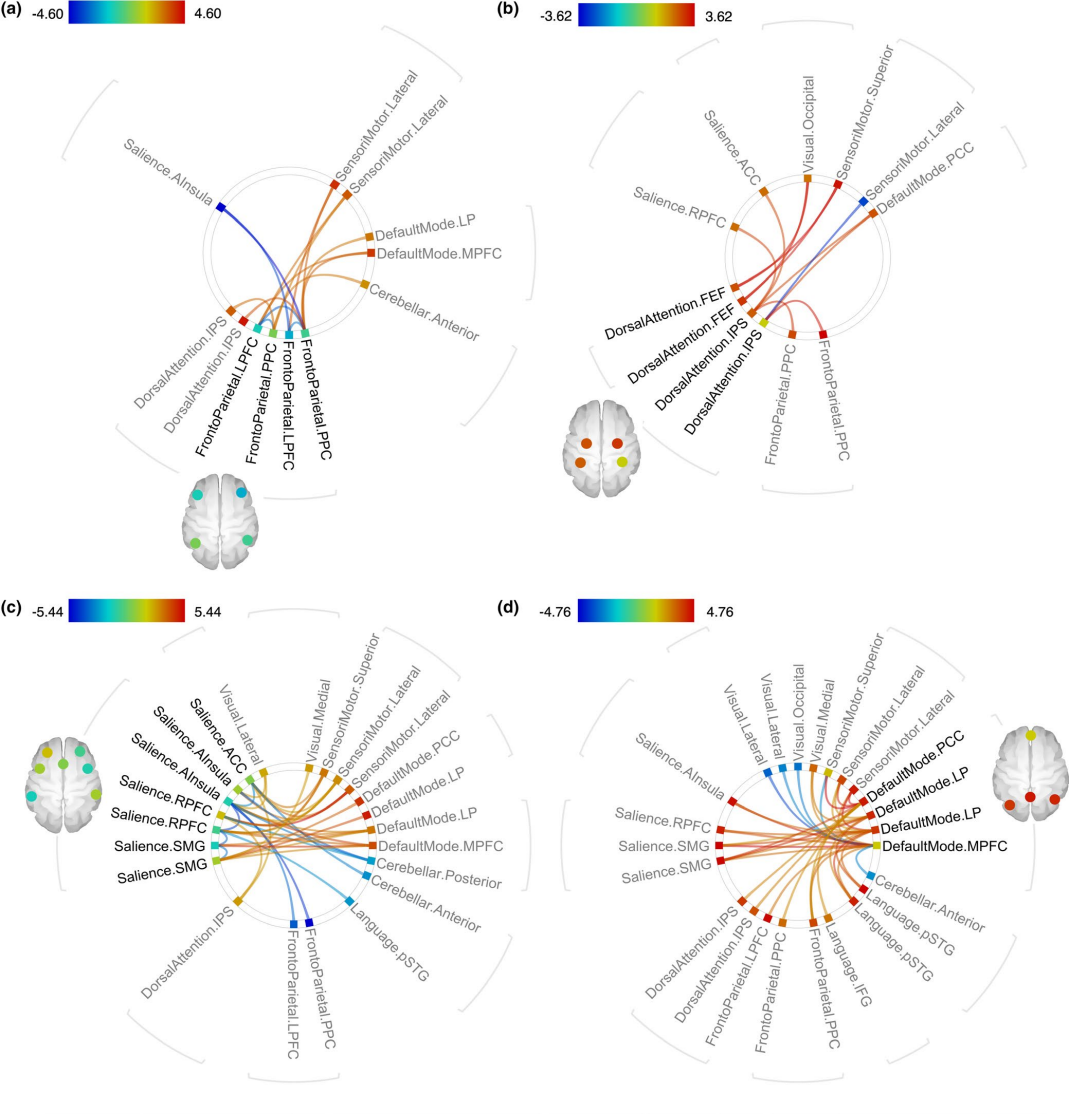
Connectograms displaying connections with significantly higher (warm colors) or lower (cool colors) connectivity for older compared with younger adults during incongruent trials with (a) frontoparietal network seeds, (b) dorsal attention network seeds, (c) salience network seeds, and (d) default mode network seeds. *p*‐value FDR‐corrected < 0.05

The results of the main analysis comparing the older and younger groups in terms of functional connectivity between network ROIs are provided in Table 4A‐D and Figure 4.

**TABLE 4:** **A‐D**. ROI‐to‐ROI functional connectivity analyses with seeds in A) the frontoparietal, B) the dorsal attention, C) the salience, and D) the default mode network, comparing older versus younger adults

**(A) Frontoparietal network seeds**		**Cohen's *d***	***t (df 58)***	***p*‐uncorr**.	***p*‐FDR‐corr**.
Seed: FPN; Lateral prefrontal cortex, R					
SMN	Lateral, R	0.88	3.35	0.0014	0.0183
*Salience*	*Anterior insula, R*	*−0.93*	*−3.56*	*0.0007*	*0.0136*
*FPN*	*Posterior parietal cortex, R*	*−0.74*	*−2.82*	*0.0066*	*0.0409*
Seed: FPN; Lateral prefrontal cortex, L					
DMN	Medial prefrontal cortex	0.82	3.12	0.0028	0.0273
SMN	Lateral, L	0.81	3.07	0.0033	0.0286
*FPN*	*Posterior parietal cortex, R*	*−0.84*	*−3.18*	*0.0023*	*0.0239*
*FPN*	*Posterior parietal cortex, L*	*−0.77*	*−2.95*	*0.0046*	*0.0372*
Seed: FPN; Posterior parietal cortex, R		0.00			
DAN	Intraparietal sulcus, R	0.90	3.44	0.0011	0.0164
DMN	Medial prefrontal cortex	0.86	3.29	0.0017	0.0197
SMN	Lateral, R	0.86	3.26	0.0018	0.0208
DMN	Lateral parietal, L	0.76	2.91	0.0051	0.0377
*Salience*	*Anterior insula, R*	*−1.21*	*−4.60*	*<0.0001*	*0.0019*
*FPN*	*Lateral prefrontal cortex, L*	*−0.84*	*−3.18*	*0.0023*	*0.0239*
*FPN*	*Lateral prefrontal cortex, R*	*−0.74*	*−2.82*	*0.0066*	*0.0409*
Seed: FPN; Posterior parietal cortex, L					
DAN	Intraparietal sulcus, L	0.81	3.08	0.0032	0.0286
Cerebellar	Anterior	0.77	2.93	0.0049	0.0377
SM	Lateral, L	0.75	2.86	0.0058	0.0395
*FPN*	*Lateral prefrontal cortex, L*	*−0.77*	*−2.95*	*0.0046*	*0.0372*

**(B) Dorsal attention network seeds**		**Cohen's *d***	***t (df 58)***	***p*‐uncorr**.	***p*‐FDR‐corr**.
Seed: DAN; Frontal eye fields, R					
SMN	Superior	0.92	3.49	0.0009	0.0152
Visual	Occipital	0.89	3.39	0.0013	0.0169
Seed: DAN; Frontal eye fields, L					
SMN	Superior	0.95	3.62	0.0006	0.0120
Visual	Occipital	0.89	3.38	0.0013	0.0169
Seed: DAN; Intraparietal sulcus, R					
FPN	Posterior parietal cortex, R	0.90	3.44	0.0011	0.0164
DMN	Posterior cingulate cortex	0.76	2.89	0.0055	0.0380
*SMN*	*Lateral, L*	*−0.82*	*−3.11*	*0.0029*	*0.0277*
Seed: DAN; Intraparietal sulcus, L					
FPN	Posterior parietal cortex, L	0.81	3.08	0.0032	0.0286
Salience	Rostral prefrontal cortex, L	0.77	2.92	0.0050	0.0377
Salience	Anterior cingulate cortex	0.75	2.84	0.0062	0.0398
DMN	Posterior cingulate cortex	0.75	2.84	0.0063	0.0398

**(C) Salience network seeds**		**Cohen's d**	***t (df 58)***	**p‐uncorr**.	**p‐FDR‐corr**.
Seed: Salience; Anterior cingulate cortex					
SMN	Lateral, L	1.01	3.85	0.0003	0.0092
SMN	Superior	0.91	3.47	0.0010	0.0156
SMN	Lateral, R	0.75	2.85	0.0060	0.0398
DAN	Intraparietal sulcus, L	0.75	2.84	0.0062	0.0398
*Cerebellar*	*Anterior*	*−0.85*	*−3.22*	*0.0021*	*0.0235*
*Salience*	*Anterior insula, R*	*−0.81*	*−3.07*	*0.0032*	*0.0286*
Seed: Salience; Anterior insula, R					
DMN	Medial prefrontal cortex	1.14	4.35	0.0001	0.0028
DMN	Lateral parietal, L	0.86	3.29	0.0017	0.0197
Visual	Lateral, R	0.75	2.84	0.0063	0.0398
SMN	Lateral, R	0.72	2.73	0.0084	0.0488
*FPN*	*Posterior parietal cortex, R*	*−1.21*	*−4.60*	*<0.0001*	*0.0019*
*Salience*	*Rostral prefrontal cortex, R*	*−1.16*	*−4.40*	*<0.0001*	*0.0028*
*FPN*	*Lateral prefrontal cortex, R*	*−0.93*	*−3.56*	*0.0007*	*0.0136*
*Cerebellar*	*Posterior*	*−0.88*	*−3.34*	*0.0015*	*0.0183*
*Salience*	*Anterior cingulate cortex*	*−0.81*	*−3.07*	*0.0032*	*0.0286*
Seed: Salience; Anterior insula, L					
SMN	Lateral, L	0.93	3.54	0.0008	0.0142
Visual	Lateral, R	0.77	2.92	0.0049	0.0377
*Cerebellar*	*Posterior*	*−1.00*	*−3.79*	*0.0004*	*0.0106*
Seed: Salience; Rostral prefrontal cortex, R					
DMN	Medial prefrontal cortex	0.96	3.66	0.0006	0.0115
DMN	Lateral parietal, L	0.89	3.40	0.0012	0.0169
SMN	Superior	0.89	3.38	0.0013	0.0169
SMN	Lateral, R	0.73	2.77	0.0074	0.0441
*Salience*	*Anterior insula, R*	*−1.16*	*−4.40*	*<0.0001*	*0.0028*
*Salience*	*Supramarginal gyrus, L*	*−0.98*	*−3.73*	*0.0004*	*0.0115*
*Language*	*Posterior supratemporal gyrus, L*	*−0.76*	*−2.88*	*0.0055*	*0.0380*
Seed: Salience; Rostral prefrontal cortex, L					
SMN	Lateral, L	1.43	5.44	<0.0001	0.0007
SMN	Superior	0.97	3.71	0.0005	0.0115
SMN	Lateral, R	0.84	3.21	0.0022	0.0235
DAN	Intraparietal sulcus, L	0.77	2.92	0.0050	0.0377
*Cerebellar*	*Posterior*	*−0.76*	*−2.89*	*0.0055*	*0.0380*
Seed: Salience; Supramarginal gyrus, R					
DMN	Lateral parietal, R	1.08	4.13	0.0001	0.0046
DMN	Posterior cingulate cortex	1.01	3.86	0.0003	0.0092
DMN	Medial prefrontal cortex	0.84	3.20	0.0022	0.0236
DMN	Lateral parietal, L	0.80	3.05	0.0034	0.0294
Visual	Medial	0.74	2.81	0.0067	0.0409
*Salience*	*Supramarginal gyrus, L*	*−0.92*	*−3.49*	*0.0009*	*0.0152*
Seed: Salience; Supramarginal gyrus, L					
DMN	Medial prefrontal cortex	1.25	4.75	<0.0001	0.0019
DMN	Lateral parietal, L	0.96	3.66	0.0005	0.0115
*Salience*	*Rostral prefrontal cortex, R*	*−0.98*	*−3.73*	*0.0004*	*0.0115*
*Salience*	*Supramarginal gyrus, R*	*−0.92*	*−3.49*	*0.0009*	*0.0152*

**(D) Default mode network seeds**		**Cohen's *d***	***t (df 58)***	***p*‐uncorr**.	***p*‐FDR‐corr**.
Seed: DMN; Medial prefrontal cortex					
Salience	Supramarginal gyrus, L	1.25	4.75	<0.0001	0.0019
Salience	Anterior insula, R	1.14	4.35	0.0001	0.0028
Salience	Rostral prefrontal cortex, R	0.96	3.66	0.0006	0.0115
FPN	Posterior parietal cortex, R	0.86	3.29	0.0017	0.0197
Salience	Supramarginal gyrus, R	0.84	3.20	0.0022	0.0236
FPN	Lateral prefrontal cortex, L	0.82	3.12	0.0028	0.0273
Language	Posterior supratemporal gyrus, L	0.81	3.08	0.0032	0.0286
SMN	Lateral, L	0.79	2.99	0.0041	0.0340
*Visual*	*Lateral, R*	*−0.97*	*−3.69*	*0.0005*	*0.0115*
*Visual*	*Occipital*	*−0.76*	*−2.91*	*0.0051*	*0.0377*
*Visual*	*Lateral, L*	*−0.74*	*−2.81*	*0.0068*	*0.0411*
*Cerebellar*	*Anterior*	*−0.74*	*−2.80*	*0.0068*	*0.0411*
Seed: DMN; Lateral parietal, R					
Salience	Supramarginal gyrus, R	1.08	4.13	0.0001	0.0046
Language	Posterior supratemporal gyrus, R Rright	0.96	3.65	0.0006	0.0115
Visual	Medial	0.79	2.99	0.0040	0.0340
Language	Posterior supratemporal gyrus, L	0.76	2.89	0.0055	0.0380
SMN	Lateral, R	0.72	2.76	0.0076	0.0449
Seed: DMN; Lateral parietal, L					
SMN	Lateral, L	1.21	4.60	<0.0001	0.0019
Language	Posterior supratemporal gyrus, L	1.07	4.06	0.0002	0.0056
Salience	Supramarginal gyrus, L	0.96	3.66	0.0005	0.0115
Salience	Rostral prefrontal cortex, R	0.89	3.40	0.0012	0.0169
Language	Posterior supratemporal gyrus, R Rright	0.89	3.40	0.0012	0.0169
Salience	Anterior insula, R	0.86	3.29	0.0017	0.0197
Salience	Supramarginal gyrus, R	0.80	3.05	0.0034	0.0294
FPN	Posterior parietal cortex, R	0.76	2.91	0.0051	0.0377
Visual	Medial	0.76	2.89	0.0054	0.0380
Seed: DMN; Posterior cingulate cortex					
SMN	Superior	1.25	4.76	<0.0001	0.0019
SMN	Lateral, L	1.20	4.58	<0.0001	0.0019
SMN	Lateral, R	1.10	4.20	0.0001	0.0042
Salience	Supramarginal gyrus, R	1.01	3.86	0.0003	0.0092
DAN	Intraparietal sulcus, R	0.76	2.89	0.0055	0.0380
DAN	Intraparietal sulcus, L	0.75	2.84	0.0063	0.0398
Language	Inferior frontal gyrus, L	0.72	2.73	0.0085	0.0488

Positive *t*‐statistics represent increased connectivity between the given ROIs in the older group compared to those in the younger group; conversely, negative *t‐*statistics signify increased connectivity in the younger group compared to than in the older group. To facilitate readability, positive *t*‐statistics are presented first in the list under each seed, and negative *t*‐statistics are printed in italics. *p*‐value analysis‐level FDR‐corrected < .05.

Abbreviations: DAN, dorsal attention network; DMN, default mode network; FDR, false discovery rate; FPN, frontoparietal network; L, left hemisphere; R, right hemisphere; SMN, sensorimotor network.

Broadly, older adults displayed relatively stronger (more positive) correlations with the DMN and sensorimotor network (SMN) both for the FPN and salience network seeds in the incongruent task condition. There was also a tendency toward stronger connections between the DAN and the FPN and salience networks. Conversely, the younger adults presented stronger connections within the FPN and within the salience network and between the regions of these two networks in the incongruent condition. The connectivity differences between the groups remained significant even after correcting for sex and education level.

**FIGURE 5:**
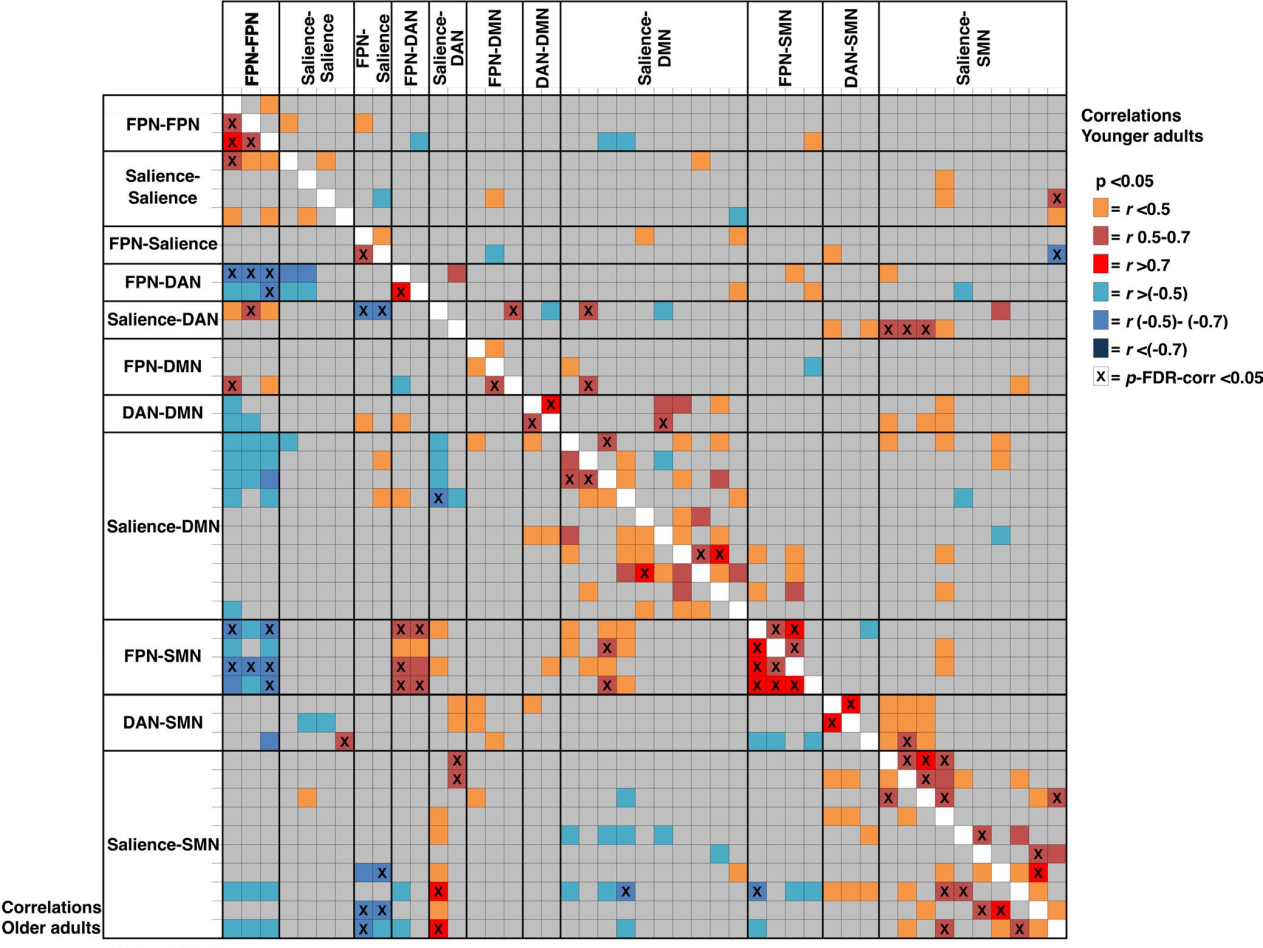
Correlations between the connectivity measures that were significantly different between age groups, within and between the frontoparietal, dorsal attention and salience networks and between these networks and the default mode and sensorimotor networks. Correlations within the younger group and within the older group are shown above and below the diagonal, respectively. Nominally significant correlations are color‐coded, and FDR‐corrected significant correlations are marked with an X

To further explore the connections that were identified in the main analysis as having significantly different strengths *between* the younger and older groups, supplementary correlation analyses were performed for the strength of these connections *within* each age group. To limit the number of statistical tests, the connectivity measures were averaged for the significant connections within the FPN, within the salience network, between the salience, FPN and DAN, and between these networks and the DMN and SMN. This was done by averaging the connectivity measures across the different seed and target regions of these networks, resulting in the 11 averaged connections displayed in Table 5 and utilized specifically for the within‐group analyses. Within the younger group, there were no correlations between the strengths of these connections and age. In the older group, the within‐network connections of the FPN and salience networks were negatively correlated with age, while the connections between the FPN and the DAN, and between the FPN and the SMN, were positively correlated with age (Table 6). For the former two within‐network connections (FPN‐FPN and salience‐salience), the correlation coefficients with age within the older group were significantly different from the homologous correlations in the younger group (*z *= −2.9, *p* = 0.002 and *z *= −2.0, *p* = 0.023, respectively). The correlations between the 11 averaged connections were also assessed, and the most prominent pattern was that in the older group, the FPN‐DAN connections and the FPN‐SMN connections were positively correlated with each other and negatively correlated with the within‐network connectivity of the FPN (Figure 5). In the younger group, there were no significant correlations between these connections after FDR corrections. When the correlation coefficients from each age group were directly compared, there was a significant interaction effect for the correlation between FPN‐SMN connections and FPN‐FPN connections (*z *= −3.0, *p* = 0.001).

**TABLE 5:** Averaged age group‐significant functional connectivity values from the main analysis

Averaged connections	Younger adults		Older adults	
FPN‐FPN	0.58	[0.53–0.63]	0.42	[0.34–0.49]
Salience‐Salience	0.46	[0.41–0.50]	0.27	[0.22–0.32]
FPN‐Salience	0.25	[0.18–0.31]	0.04	[÷0.02–0.10]
FPN‐DAN	÷0.06	[÷0.11 to ÷0.01]	0.10	[0.03–0.18]
Salience‐DAN	0.02	[÷0.03–0.06]	0.14	[0.09–0.19]
FPN‐DMN	÷0.04	[÷0.10–0.02]	0.11	[0.06–0.16]
DAN‐DMN	0.02	[÷0.05–0.08]	0.15	[0.09–0.22]
Salience‐DMN	÷0.13	[÷0.16 to ÷0.09]	0.05	[0.01–0.09]
FPN‐SMN	÷0.23	[÷0.28 to ÷0.18]	÷0.07	[÷0.14–0.001]
DAN‐SMN	0.23	[0.17–0.29]	0.30	[0.25–0.35]
Salience‐SMN	÷0.01	[÷0.05–0.03]	0.13	[0.10–0.17]

Note

The 74 ROI‐to‐ROI correlations significantly different between the age groups in the main analysis, were averaged across seeds and targets for connections between regions within the FPN, within the salience network, between the salience, FPN, and DAN, and between these networks and the DMN and SMN. These averaged functional connectivity values were only used for supplementary analyses within each age group and are shown to aid interpretation of the main results. The values are reported as mean [95%‐confidence interval for the mean].

Abbreviations: DAN, dorsal attention network; DMN, default mode network; FPN, frontoparietal network; SMN, sensorimotor network.

**TABLE 6:** Correlation between age and connectivity measures within the older group

Connections	Pearson's correlation *r*	*p*
FPN‐FPN	−0.69	<0.001*
Salience‐Salience	−0.48	0.007*
FPN‐Salience	0.11	0.558
FPN‐DAN	0.57	0.001*
Salience‐DAN	−0.40	0.030
FPN‐DMN	−0.39	0.036
DAN‐DMN	0.37	0.045
Salience‐DMN	0.34	0.070
FPN‐SMN	0.60	<0.001*
DAN‐SMN	−0.20	0.290
Salience‐SMN	−0.28	0.139

Note

Significant correlations after false discovery rate correction for multiple comparisons (*p* < 0.05) are asterisked.

Abbreviations: DAN, dorsal attention network; DMN, default mode network; FPN, frontoparietal network; SMN, sensorimotor network.

The averaged connectivity measures were used in further supplementary analyses to assess the relationship with eStroop task performance within each age group. Connectivity was not related to mean RT, ACC, or RT interference effect within either age group. In the younger group, there were nominally significant weak correlations between the ACC interference effect and the connectivity between the FPN and the DAN (Spearman's ρ: −0.44, *p* = 0.015) and between the FPN and the DMN (Spearman's ρ: 0.38, *p* = 0.039), but these would not survive more stringent correction for multiple testing. No correlations were found within the older group. Similarly, no correlations between connectivity and score on the trail‐making test A, used as a separate measure of processing speed, survived correction for multiple comparisons (FDR‐corrected *p*‐value < 0.05).

To explore other factors that could possibly impact the connectivity differences observed between the groups, modified Framingham vascular risk score (FVRS), Fazekas’ score, and total white matter volume (WMV) were separately considered as covariates into the GLMs for the connectivity measures (not averaged) as a function of age group. The FVRS was a statistically significant covariate for functional connectivity strength in nine of the 74 age‐sensitive connections (Supporting Information, Table S4A) in the same direction as age. Entering the FVRS in the model reduced the regression coefficient for age group slightly, but it remained significant overall. Fazekas’ score was only a significant covariate in the models of two of the functional connections, again in the same direction as age group (Supporting Information, Table S4B). When taking into account Fazekas’ score, the impact of age group on intra‐salience network connectivity between the left and the right supramarginal gyrus was reduced to the half, and it was no longer statistically significant. For the models with and without inclusion of WMV (Supporting Information, Table S4C), the relationship between the two independent variables (age group and WMV) was more complex. Total WMV was a significant predictor of functional connectivity with the same directionality as age for some connections, including connectivity between the posterior parietal cortex of the FPN and the intraparietal sulcus of the DAN. WMV was also a significant dependent factor of functional connectivity for connections between the salience network and the SMN, but in the reverse direction to that of age. Nevertheless, in all the models where WMV was a significant predictor, the differences in connectivity between groups remained statistically significant even after correcting for WMV.

Total gray matter volume (GMV) was not included as a covariate in the between‐group analyses because of the high degree of multicollinearity with age group. In within‐group regression analyses of functional connectivity as a function of age, total GMV in cm^3^ was a nominally significant predictor of within‐network connectivity in the FPN (standardized beta: 0.28, *p* = 0.046, *R* ^2^ = 0.54) and within‐network connectivity in the salience network (beta: 0.37, *p* = 0.027, *R* ^2^ = 0.36) in the older group, and within‐network connectivity in the FPN (beta: −0.42, *p* = 0.040, *R* ^2^ = 0.15) and connectivity between the FPN and salience networks (beta: −0.44 , *p* = 0.029, *R* ^2^ = 0.17) in the younger group. The GMV associations did not remain significant after FDR correction. Notably, age remained a significant predictor of the connectivity measures within the older group for the models where GMV was a significant predictor.

## 4 DISCUSSION

The results of the emotional face–word Stroop task employed in the current study demonstrated a robust and reliable Stroop interference effect in terms of both response accuracy and RT. Although the older participants were slower to respond and made more mistakes, there was no significant difference in the interference effect between the age groups. Previous studies of face–word emotional Stroop tasks in older and younger adults have reported conflicting results. Agusti et al. (2017) found an increased emotional Stroop effect for identification of emotional faces with word distractors (but not vice versa) in older participants. In their study, the interference effect was calculated as the absolute RT difference between congruent and incongruent trials, a method that is believed to overestimate interference differences between age groups by failing to control for age‐related differences in processing speed (Verhaeghen, 2011). Indeed, when we calculated the absolute RT interference effect, there did appear to be a trend toward an age‐related difference, but it disappeared entirely when the differences in baseline RTs for the congruent trials were accounted for. In a recent study where the effect of general slowing on RTs was considered, Berger et al. (2019) found no significant increase in RT emotional Stroop face–word interference effect between older and younger adults. It should be noted that while both of these previous studies and our study included faces expressing happiness, the negative emotional valence varied—Agustí et al. used sadness, Berger et al. anger, while we chose to study faces expressing fear. None of the three studies, however, found any significant interaction between emotional valence and age in terms of RTs. Even though the sample sizes were relatively small (60 participants in each), our results, combined with those of Berger et al., strengthen the view that there is no true age‐related change in RT emotional face–word Stroop interference effect.

Comparing RTs and accuracy scores, there was no evidence that younger and older adults employed different strategies for solving the task in terms of speed/accuracy trade‐offs. This is in line with what Waring et al. found using an emotional Go/No‐Go task to compare younger and older adults (Waring et al., 2019). They also reported greater emotional response inhibition (fewer false alarms) in older than in younger individuals. Participants in our older group did make more mistakes in general, but no significant age‐related difference was detected in either accuracy interference effect or accuracy dependent on emotional valence. In the previous studies on the emotional Stroop face–word task in aging, Agustí et al., like us, reported lower accuracy in the older group (Agusti et al., 2017). Berger et al., on the other hand, found higher accuracy in the older group than the in young group when using a task version with words describing the emotion (e.g., “happy,” ”angry,” “neutral” written across the face), but no main difference in accuracy between the age groups in a similar task with emotional words (e.g., “thrill,” “abuse,” “bench” superimposed on the face; Berger et al., 2019). Their results emphasize the potential impact of subtle variations in stimulus sets. In all studies, the accuracy scores were generally high, and measurements of accuracy, as opposed to RTs, could be influenced by a ceiling effect. In a working memory task study with emotional distractors, Ziaei et al. found that older adults had worse working memory accuracy for emotional versus neutral distractors, but no such accuracy difference was observed for younger adults, whereas for RTs, there was no age group by condition interaction (Ziaei et al., 2018). It is possible that older adults employ so much attentional resources to overcome emotional distraction without excessive RT or errors that attention for encoding the information is restricted, resulting in reduced working memory performance.

Contrasting incongruent and congruent trials across all participants, we replicated the results of previous fMRI studies that used comparable emotional Stroop paradigms for whole‐brain analyses of mostly young, healthy control participants (Chechko et al., 2009, 2012, 2013; Chen et al., 2018; Fleury et al., 2014; Rey et al., 2014; Song et al., 2017)—showing involvement of the ventrolateral prefrontal cortex/inferior frontal gyrus, primary and supplementary motor areas, insula, middle temporal cortex, inferior parietal lobule, and fusiform gyrus in emotional face–word interference. As fMRI brain activation differences between younger and older adults during this task had not been explored before, we decided to conduct a whole‐brain analysis for comparing the age groups rather than making specific hypotheses about the localization of potential age‐related differences. No brain areas displayed significantly different activation between the two groups. A supplementary and explicitly exploratory analysis with an uncorrected *p* < 0.001 revealed bilateral clusters of relatively higher activity in the inferior frontal gyrus in the older group compared to that in the younger group for the incongruent versus congruent contrast. The location of this investigative finding is noteworthy, as two activation likelihood estimation meta‐analyses comparing cognitive and emotional interference processing have shown that while activation in these two domains of interference map onto largely overlapping brain areas, foci in the inferior frontal gyrus appear to be distinct for emotional interference (Chen et al., 2018; Hung et al., 2018). Left inferior frontal gyrus activation was also found to be linked to better performance in a working memory task with emotional distractors, where older adults also activated frontal areas more than younger adults for emotional versus neutral distractors (Ziaei et al., 2018). Contrarily, in a study using a modified Eriksen Flanker task, there was a tendency toward less activation of the inferior frontal gyrus in older adults for emotional relative to non‐emotional interference (Samanez‐Larkin et al., 2009). It has been theorized that emotional distractors impact performance by disrupting the balance of activity between a “dorsal executive system” (mainly involved in cognitive, non‐emotional control and associated with the FPN) and a “ventral affective system” (tied to emotional processing and overlapping in part with the salience network in addition to canonical affective regions such as the amygdala; Bush et al., 2000; Iordan & Dolcos, 2017). Within this framework, one could hypothesize that older adults over‐activate frontal areas in order to successfully resolve emotional conflict.

Increased prefrontal activity is a recurrent finding in many, but not all (Oren et al., 2017; Schweizer et al., 2019), fMRI studies comparing older and younger adults. The observation that older adults tend to have higher frontal activity and lower occipital activity forms the basis for the “posterior‐anterior shift in aging” model first proposed by Grady et al. (Davis et al., 2008; Grady et al., 1994). For emotion processing, a similar model assuming that older adults recruit frontal areas more and the amygdala less than younger adults has been named the “frontoamygdalar age‐related difference in emotion model” (St Jacques et al., 2009). This age‐related pattern has been observed in explicit, but not implicit, negative emotion processing (Zsoldos et al., 2016). Whether this increase in prefrontal activity indicates a compensatory mechanism, neuroadaptation, or loss of neural specificity has been a subject of inquiry (Lloyd et al., 2021; Morcom & Henson, 2018; Myrum, 2019). In our study, there was a trend toward higher inferior frontal gyrus activity being related to higher accuracy in the emotional Stroop task for older adults (Supporting Information, Table S3). This could suggest that an activity increase would be compensatory. Finding increased BOLD signal activity, however, cannot be straightforward interpreted as increased neuronal activity. Mohtasib et al. studied a color–word Stroop task in adults (aged 18–71 years) while simultaneously acquiring BOLD signal and using arterial spin labeling to measure cerebral blood flow (Mohtasib et al., 2012). The study found an age‐related increase in BOLD signal with the greatest increase in the medial frontal gyri, although the cerebral blood flow was unchanged. The authors attributed the BOLD signal increase to a reduction in oxygen metabolism and hence an age‐related decrease in neural activity. This emphasizes the need to consider potential changes in the neurovascular coupling in studies on fMRI and aging. In the current study, we rescaled the BOLD signal in the eStroop task using the RSFA. The case for this scaling approach is supported by the report that variation in RSFA with age is significantly mediated by vascular factors and not by neuronal activity as measured using magnetoencephalography (Tsvetanov et al., 2015). Rescaling, however, did not substantially change the results, and no significant voxel‐wise activity difference between the age groups remained after FWE‐correction. Standard cluster‐extent‐based thresholding would yield the same result. Future studies could specifically assess activity changes within the inferior frontal gyrus, but the results of the current study suggest that the basic brain activation pattern seen in young healthy adults during emotional Stroop interference processing is largely preserved with aging.

The network dedifferentiation theory of aging posits that large‐scale networks change with age, becoming less segregated and more integrated (Geerligs et al., 2015). Previous resting‐state and cognitive task fMRI studies have found support for this theory by observing increased between‐network connectivity and decreased within‐network connectivity in older adults relative to younger adults (Damoiseaux, 2017; Grady et al., 2016), but it is unknown whether this also pertains to emotional tasks. In the current study, we were able to replicate these findings for an emotional conflict task. Previous fMRI studies of connectivity measures during emotional tasks in groups with varying adult age have mainly focused on the connectivity of the amygdala and prefrontal cortex (Cook et al., 2007; Ritchey et al., 2011; St Jacques et al., 2010; Winecoff et al., 2011; Ziaei et al., 2017). Some resting‐state fMRI studies have assessed broader network connectivity in aging in the context of emotion processing. Nashiro et al. reported disrupted resting functional connectivity in cognitive networks with age, but preservation of emotional networks (Nashiro et al., 2017). Lyoo and Yoon classified individuals as “emotionally older” if they displayed a higher recognition of positive emotions relative to negative emotions (i.e., a positivity effect of aging) and discovered that resting‐state connectivity between the executive control network and the DMN was greater in the “emotionally older” subjects (Lyoo & Yoon, 2017). We now present emotional task fMRI data of network connectivity that resemble the pattern seen in non‐emotional cognitive tasks. A central observation in previous studies of brain network organization is the anticorrelation between cortical networks activated by attention‐demanding tasks and the DMN, which is known to be active during rest. Within the field of aging‐related research, the anticorrelation between the DAN and DMN has been most widely studied, and the results converge on a reduction in DAN‐DMN‐anticorrelation with age (Dorum et al., 2016; Spreng et al., 2016). In line with this observation, we also found that older adults had stronger connections between the DAN and DMN during the eStroop task, compared to their younger counterparts. The same was also true for the FPN and the salience network, as both had stronger (more positively correlated) connections with the DMN in the older adults during interference processing. The FPN‐DAN and salience network‐DAN connections were also relatively stronger, while the salience network‐FPN connections were weaker in the older adults. All three of these putatively domain‐specific attention/control networks have similarities with an overarching, more general‐domain network called the extrinsic mode network (EMN; Hugdahl et al., 2015). These broad brain networks appear to be dynamically up‐ and down‐regulated in response to task demands (Hugdahl et al., 2019). The connectivity results from the current and previous studies suggest that this dynamic changes with aging. What could be the underlying causes of this functional reorganization with age? The degree of anticorrelation between DMN and DAN during task was shown by Avelar‐Pereira et al. to be associated with resting cerebral blood flow in the DMN. As older adults had lower gray matter cerebral blood flow, this was interpreted as indicating that lower DMN activity at rest underlies the age‐related deficit in anticorrelation during tasks (Avelar‐Pereira et al., 2017). Disrupted within‐DMN connectivity at rest is probably the most consistent finding in studies of brain aging (Damoiseaux, 2017). Interestingly, the DMN has also been found to be the location of the earliest amyloid accumulations in the preclinical development of Alzheimer's disease (Palmqvist et al., 2017). These first manifestations of amyloid deposition are also associated with functional connectivity changes in the DMN. This occurs at a stage before other biomarkers, such as glucose hypometabolism and atrophy, are present and many years before clinical signs of the disease may manifest. Very early amyloid pathology could, therefore, contribute to age‐dependent alterations in the EMN‐DMN anticorrelation also in otherwise healthy older adults.

To what degree do the reported network connectivity alterations depend on age‐associated vascular factors and atrophy? We did not correct for vascular reactivity in the connectivity analyses, but this was done by Avelar‐Pereira et al. in their study of non‐emotional interference, wherein the pattern of age‐related connectivity alterations remained intact (Avelar‐Pereira et al., 2017). In the present study, we included a clinical cardiovascular disease risk score and Fazekas’ score, a crude but easily accessible measure of white matter lesion load commonly attributed to small vessel disease (Fazekas et al., 1987), as covariates. This did not alter the main connectivity differences between the older and younger groups, indicating that the findings were not highly reliant on cerebrovascular disease features. All functional connectivity must have some structural correlate. Age‐related decline in executive functions was in a longitudinal study associated with functional connectivity, but only explained by structural connectivity (Fjell et al., 2017). The most prominent structural feature of advancing brain aging is atrophy. In our study, age‐related connectivity changes remained after adjustment for white matter and gray matter volume. In the older adult group, the relative reduction in within‐FPN connectivity was anticorrelated with increase in FPN‐DAN and FPN‐SMN connectivity during the eStroop task, suggesting that these opposing age‐related processes are linked. We propose that this shift is a feature of the aging process, partly independent of vascular changes and general atrophy.

A much‐discussed question is whether changes in functional network interactions with age are compensatory in nature (Cabeza et al., 2018). The reasoning is that the aging brain, faced with a decline in processing speed and other cognitive domains, recruits additional brain areas and networks in order to still successfully process tasks. Alternatively, the age‐related changes with increased cross‐talk between networks could be underlying the increased processing speed. We found no definite support for this in the current study, as the connectivity changes were not associated with either eStroop task performance or processing speed. In a non‐emotional multi‐source interference task, increased DMN‐DAN anticorrelation from rest to task has been shown to correlate with better interference resolution, suggesting that the reduction in anticorrelation (presumably reduction in task‐related DMN suppression with age) does not appear to represent successful compensation (Avelar‐Pereira et al., 2017). Network segregation (as opposed to dedifferentiation) in lifespan resting‐state data has also been shown to be predictive of superior long‐term episodic memory, independent of age (Chan et al., 2014). On the other hand, the functional connectivity between the DMN and attentional networks has also been shown to increase in response to rising task complexity (higher demand for cognitive control), which has been related to better task performance in young adults (O'Connell & Basak, 2018). Moreover, the relationship between co‐activation of the DMN during execution of cognitive tasks and task performance appears to be task‐specific and possibly age‐related. In the subsequent memory paradigm, younger adults tend to have greater deactivation of the DMN during encoding of the stimuli they later remember as opposed to the stimuli they forget. The opposite seems to occur in older adults, with reduced DMN deactivation for the remembered versus forgotten stimuli (Maillet & Schacter, 2016). Other studies have similarly found that the age‐related differences in network interactions assessed by functional connectivity are partly contingent on the specific cognitive task (Archer et al., 2016; Geerligs et al., 2015); however, our understanding is limited by the low number of tasks that have been examined to date (Hughes et al., 2020). Emotional tasks could be a unique case, as performance seems more resilient to the age‐related deterioration observed in other cognitive domains. As we detected no age‐related difference in interference effect for the eStroop task, it is possible that our healthy older participants used their greater involvement of the DMN (shown by the more positive correlation of activity, or even reversal of anticorrelation, between regions of the DMN and the FPN and between regions of the DMN and the salience network) to maintain normal emotional conflict processing. Indeed, evidence from resting‐state fMRI data implicates the DMN in emotional processing and emotion regulation (Pan et al., 2018). An fMRI study of the two emotion regulation strategies, distraction and reappraisal, found that while there was no age‐related distinction in terms of the memory of the presented stimuli, younger adults had a more segregated brain activation pattern, with disengagement of the posterior medial cortex during reappraisal relative to distraction. Older adults, meanwhile, engaged this posterior part of the DMN to the same extent for both emotion regulation strategies (Martins et al., 2015). Interestingly, middle‐aged offspring from long‐lived families, presumably individuals with a slower pace of aging, have been shown to deactivate the medial posterior cingulate cortex more than normal aging control subjects, when performing a working memory task with emotional distractors (Oei et al., 2018). Other fMRI studies have found that aging is associated with greater involvement of the medial prefrontal cortex, corresponding to the frontal part of the DMN, during emotional suppression (Katsumi et al., 2020) and viewing of emotionally negative scenes (van Reekum et al., 2018). Martins and Mather have also proposed that the increased connectivity between the DMN and attentional and executive control networks supports improved emotion regulation in later life (Martins & Mather, 2016). The DMN is known to be active during internally directed cognition and self‐generated thoughts (Andrews‐Hanna et al., 2014) and has been suggested to play a role in automated, overlearned responses (Vatansever et al., 2017). One interpretation is that older adults make more use of self‐referential information and pre‐existing schemas for the processing and control of emotional information.

In what way could the results of the current study contribute to our understanding of late‐life affective disorders? Healthy aging is not linked to mood problems, while people describe declining physical and cognitive function with advancing age, self‐reported mental well‐being and stability actually improves (Jeste et al., 2013; L. M. Williams et al., 2006). This positive age‐related effect of emotional well‐being has even been shown to persist during the current COVID‐19 pandemic (Carstensen et al., 2020). According to the socioemotional selectivity theory, this emotional paradox of aging is explained by a motivational shift—when the perceived remaining time in life is limited, people tend to prioritize current and emotionally meaningful goals to maximize life satisfaction (Carstensen et al., 1999). This theory also explains the “positivity effect of aging” as seen in some attention and memory tasks. Using resting‐state fMRI, this positivity effect has previously been linked to increased connectivity between the DMN and the FPN (Lyoo & Yoon, 2017). However, the positivity effect is only present when cognitive resources are relatively abundant. The effect disappears or even reverses (to a bias toward negative information) if cognitive reserves are depleted or attention is divided (Reed & Carstensen, 2012). If the normal performance of our older participants in the eStroop task was in part dependent on the observed greater functional connectivity between attentional/executive control networks and the DMN and SMN, this greater involvement of multiple networks might in itself take up capacity, in keeping with the previous finding that greater cross‐talk between these networks is seen in response to increased task complexity. This makes for a vulnerable system. Known risk factors for late‐life depression, such as social stress, sleep disturbance, or pain (Chang et al., 2016), may all occupy attentional resources, resulting in the reversal of the positivity effect. Mood and perceived stress have also been shown to alter the association between aging and the extent of task‐related DMN deactivation (Soares et al., 2017). Moreover, stress appears to enhance the neural reactivity to emotional faces selectively in older adults (Everaerd et al., 2017). It is well established that deficits in attention and inhibition of irrelevant emotional information are prevalent in late‐life depression (Korsnes & Ulstein, 2014). The structural connections between these networks, particularly between the posterior regions of the DMN and the frontal areas of the attention networks, are also vulnerable to pathological events that become more common with advancing age. Such events include aging effects of protein dysmetabolism such as amyloid pathology, microstructural changes, and major cerebrovascular events, all of which have been associated with late‐life depression (Alexopoulos, 2019). Functional interactions of large‐scale networks during emotional interference in patients with late‐life depression relative to those with healthy aging should be addressed in future studies.

The current study has certain limitations. In‐scanner movement is a ubiquitous challenge in fMRI studies, and in our study, we recorded more movement in the older group. Interestingly, movement was related to task performance in the young, but not in the older participants. Realigning images across scanning time and including motion parameters in the analyses counteract the effects of movement, although not completely, and we cannot exclude the possibility that residual motion‐related artifacts may have influenced the results. Another clear limitation is that we only included positive and negative emotional stimuli and no neutral comparison in the task. This made it difficult to assess the direction of effects based on emotional valence and prevents us from making statements about the presence or absence of an age‐related positivity effect (Reed & Carstensen, 2012).

There are several problems with a cross‐sectional design in the study of aging. There are multiple sources of bias when comparing measures of brain activity between younger and older adults. Vascular changes have already been mentioned. Even though we rescaled the voxel‐wise analysis for RSFA as an estimate of vascular reactivity, we could not correct for cerebral blood volume or cerebral metabolic rate of oxygen. Both these factors have been found to change with age in various animal and human studies, but the evidence is conflicting with respect to the degree and direction of the resulting impact on the BOLD signal (for a review see [Wright & Wise, 2018]). These unknown variables in the neurovascular coupling challenge the interpretation of BOLD signal discrepancies between age groups as true differences in neuronal activity. At the same time, accumulation of damage to the vascular system over time is hypothesized to be a key causal factor in the brain aging process itself and presumably contributes to functional reorganization of the aging brain in the first place. Another challenge is the known decline in peripheral sensory functions with age. For example, Porto et al. discovered that one of the best replicated age‐associated phenomena in the event‐related potentials literature, reduction in P3b amplitude, is eliminated when controlling for visual acuity (Porto et al., 2016). In the current study, normal or corrected‐to‐normal vision was listed as an inclusion criterion, but visual acuity was not formally tested. MRI‐compatible googles were used as needed, but they cannot fully compensate for potential age‐related differences in vision. The two age groups also differed in their use of medications. All participants were interviewed about their use of prescription and over‐the‐counter drugs. None of the included participants used drugs that were judged to potentially influence cognitive functions, but medication‐related effects on the results cannot be entirely ruled out. Recruiting only older adults who are totally medication free, even though possible, would create a study group of “super‐agers,” thereby reducing the generalizability of the results. Another possible source of bias is that aging is accompanied by a reduction in global and regional brain volumes and enlargement of the ventricles which can interfere with normalization to a standard brain template. This could potentially yield systematically higher levels of image pre‐processing inaccuracies in the older group. In order to overcome such between‐group biases and make stronger statements about aging effects, longitudinal studies are needed.

## 5 CONCLUSIONS

We present an fMRI study of brain activation and functional connectivity in older and younger adults during an emotional face–word Stroop task and found preserved emotional interference resolution with age. The voxel‐wise brain activation pattern during emotional Stroop task performance is largely comparable between younger and older adult groups. Compared to younger individuals, older individuals have stronger connections between major brain networks, including the DMN, during emotional interference processing, and weaker within‐network connectivity of the attention/control networks. These functional connectivity results replicate and expand on previous results of non‐emotional task fMRI studies of aging.

## CONFLICT OF INTEREST

Kenneth Hugdahl owns shares in the company NordicNeuroLab Inc. (https://nordicneurolab.com/) that produced the response collection device and LCD monitor used in the fMRI study. All authors declare that they have no conflicts of interest regarding the content of this article.

## **AUTHOR** **CONTRIBUTIONS**

Ina S. Almdahl involved in conceptualization, methodology, investigation, data curation, formal analysis, and writing the original draft. Liva J. Martinussen involved in investigation and writing, reviewing and editing. Ingrid Agartz involved in supervision and writing, reviewing and editing. Kenneth Hugdahl involved in supervision and writing, reviewing and editing. Maria S. Korsnes involved in supervision, funding acquisition, and writing, reviewing and editing.

### PEER REVIEW

The peer review history for this article is available at https://publons.com/publon/10.1002/brb3.2052

## Supporting information

Supplementary Material

## Data Availability

De‐identified fMRI scans and other data used in this study have not been made available through any open research database to date, as the current approval of the project from the Data Protection Authority at Oslo University Hospital does not allow such data sharing.
