# Supplementary material for: Inhibition of emotions in healthy aging: age‐related differences in brain network connectivity
Source: Brain Behav. 2021 Feb 4;11(5):e02052. doi: 10.1002/brb3.2052 (PMC8119855; doi:10.1002/brb3.2052)
Supplement: Supplementary file 1 — Supplementary Material [file BRB3-11-e02052-s001.docx]

Supporting Information for

*Inhibition of emotions in healthy ageing: Age-related differences in brain network connectivity*

Ina S. Almdahl, Liva J. Martinussen, Ingrid Agartz, Kenneth Hugdahl, Maria S. Korsnes

**Corresponding author**: Ina S. Almdahl

E-mail: [ina.almdahl@medisin.uio.no](mailto:ina.almdahl@medisin.uio.no)

Postal address: Oslo University Hospital, Department of Old Age Psychiatry, P.O. box 4950, 0424 Oslo, Norway

**Supplementary Table S1:** Two-sample *t*-test comparing activation during the emotional Stroop task (contrast incongruent trials > congruent trials) between older and younger adults, threshold non-parametric uncorrected *p*-value < 0.001, 10 000 permutations. The table lists statistics and MNI coordinates for local maxima, up to 3 maxima >8mm apart.

| **Brain region** | **BA** | **Side** | ***n* voxels** | ***t*** | ***Cohen’s d*** | ***p***  **FWE-corr.** | ***p***  **uncorr.** | **Peak voxel**  **(x, y, z, MNI)** | | |
| --- | --- | --- | --- | --- | --- | --- | --- | --- | --- | --- |
| **Older>Younger:** |  |  |  |  |  |  |  |  |  |  |
| Inf. frontal gyrus, pars orbitalis | 47 | R | 66 | 3.99 | 1.05 | 0.19 | 0.0002 | 44 | 28 | -4 |
| Inf. frontal gyrus, triangular part | 45 | L | 53 | 3.95 | 1.04 | 0.20 | 0.0001 | -50 | 26 | 0 |
| Supramarginal gyrus | 39 | L | 15 | 3.76 | 0.99 | 0.29 | 0.0003 | -62 | -48 | 36 |
| Inf. frontal gyrus, pars orbitalis | 10 | R | 28 | 3.64 | 0.96 | 0.35 | 0.0002 | 44 | 46 | -6 |
| M. cingulate/paracingulate gyri | 8 | R | 30 | 3.54 | 0.93 | 0.40 | 0.0003 | 8 | 26 | 34 |
| Inf. parietal gyrus | 39 | L | 19 | 3.50 | 0.92 | 0.42 | 0.0002 | -54 | -52 | 44 |
| Inf. frontal gyrus, pars orbitalis | 47 | L | 4 | 3.36 | 0.88 | 0.50 | 0.0009 | -42 | 28 | -8 |
| Inf. frontal gyrus, pars orbitalis | 47 | L | 1 | 3.25 | 0.85 | 0.56 | 0.0009 | -42 | 32 | -6 |
| **Younger>Older:** |  |  |  |  |  |  |  |  |  |  |
| Temporal pole, m. temp. gyrus | 38 | R | 3 | 3.56 | 0.93 | 0.39 | 0.0002 | 58 | 4 | -16 |

Voxel size 2mm^3^. Search volume 151 015 voxels.

**Supplementary Figure S1**: Incongruent>congruent-contrast, older>younger adults, threshold non-parametric uncorrected *p*-value < 0.001.


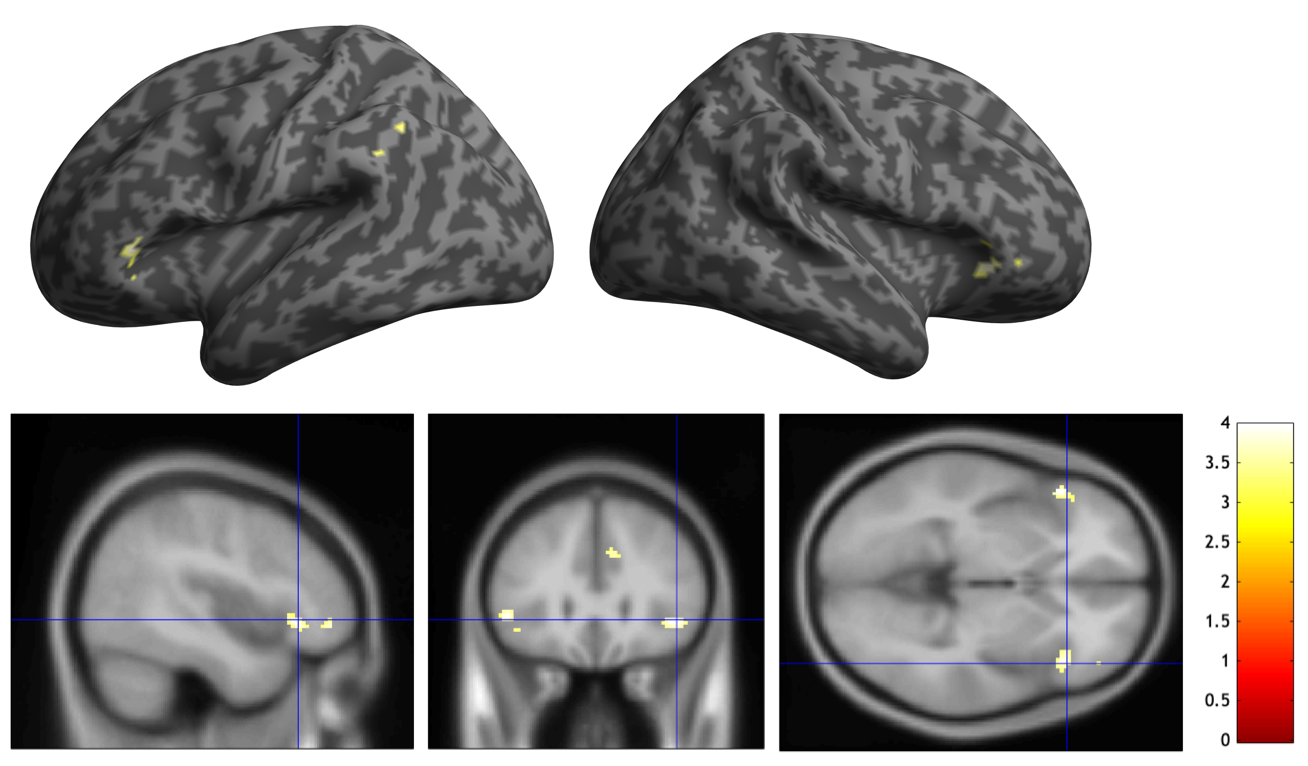


**Supplementary Table S2**: Same results as in table S1, corrected for resting-state amplitude fluctuations.

| **Brain region** | **BA** | **Side** | **n Voxels** | ***t*** | ***Cohen’s d*** | ***P***  **FWE-corr.** | ***P***  **Uncorr.** | **Peak voxel**  **(x, y, z, MNI)** | | |
| --- | --- | --- | --- | --- | --- | --- | --- | --- | --- | --- |
| **Older>Younger:** |  |  |  |  |  |  |  |  |  |  |
| Inf. frontal gyrus, pars orbitalis | 47 | L | 57 | 4.33 | 1.14 | 0.09 | 0.0002 | -48 | 24 | -4 |
| Inf. frontal gyrus, pars orbitalis | 47 | R | 66 | 4.14 | 1.09 | 0.13 | 0.0001 | 40 | 28 | -6 |
| Inf. frontal gyrus, pars orbitalis | 47 | R | 20 | 4.13 | 1.08 | 0.14 | 0.0002 | 46 | 46 | -6 |
| M. cingulate/paracingulate gyri | 8 | R | 52 | 3.77 | 0.99 | 0.28 | 0.0001 | 6 | 30 | 34 |
| Supramarginal gyrus | 39 | L | 1 | 3.46 | 0.91 | 0.43 | 0.0006 | -62 | -48 | 36 |
| Precentral gyrus | 4 | L | 1 | 3.36 | 0.88 | 0.49 | 0.0008 | -18 | -26 | 60 |
| Inf. parietal gyrus | 39 | L | 7 | 3.31 | 0.87 | 0.52 | 0.0005 | -54 | -52 | 42 |
| Inf. frontal gyrus, opercular part | 44 | L | 4 | 3.28 | 0.86 | 0.54 | 0.0007 | -52 | 12 | 4 |
| Fusiform gyrus | 37 | R | 4 | 3.26 | 0.86 | 0.55 | 0.0009 | 30 | -54 | -10 |
| Inf. frontal gyrus, triangular part | 9 | L | 1 | 3.18 | 0.84 | 0.60 | 0.0008 | -46 | 34 | 26 |
| **Younger>Older** |  |  |  |  |  |  |  |  |  |  |
| Temporal pole, m. temp. gyrus | 38 | R | 6 | 3.61 | 0.95 | 0.36 | 0.0003 | 58 | 4 | -16 |

Voxel size 2mm3. Search volume 150 977 voxels.

**Supplementary Table S3:** Bivariate correlation analyses between the beta-weights extracted from the main bilateral inferior frontal gyrus clusters shown in figure S1 and performance on the eStroop task in the two age groups (Spearman’s ρ and p-values are listed):

|  | **Younger adults** n=30 | | | **Older adults** n=30 | | |
| --- | --- | --- | --- | --- | --- | --- |
| ROI | Mean RT of correct trials | Mean ACC | Relative RT interference | Mean RT of correct trials | Mean ACC | Relative RT interference |
| Right inferior frontal gyrus | ρ -0.265  *p*=0.157 | ρ 0.255  *p*=0.174 | ρ 0.079  *p*=0.677 | ρ 0.018  *p*=0.927 | ρ 0.323  *p*=0.081 | ρ 0.333  *p*=0.072 |
| Left inferior frontal gyrus | ρ -0.149  *p*=0.431 | ρ 0.077  *p*=0.686 | ρ 0.013  *p*=0.947 | ρ -0.106  *p*=0.577 | ρ 0.364  *p*=0.048 | ρ 0.072  *p*=0.704 |

**Supporting Table S4A-C**: Model explorations for the functional connections with significantly different strength between the two age groups. The tables show details for the general linear regression models where A) modified Framingham risk score B) Fazekas’ score, C) white matter volume, were significant independent factors, respectively.

| **Table S4A: Modified Framingham vascular risk score (FVRS)** | | | | | | | |
| --- | --- | --- | --- | --- | --- | --- | --- |
| **Connection: DAN; Frontal eye fields R – SMN; Superior** | | | | | | | |
| Model 1: Age-group alone | | | | Model 2: Age-group + FRS | | | |
|  | β | p-value | r^2^ |  | β | p-value | r^2^ |
| Age-group | 0.17 | 0.001 | 0.17 | Age-group | 0.13 | 0.011 | 0.25 |
|  |  |  |  | FVRS | 0.01 | 0.024 |  |
| **Connection: DAN; Frontal eye fields L – SMN; Superior** | | | | | | | |
| Age-group | 0.18 | 0.001 | 0.19 | Age-group | 0.12 | 0.013 | 0.33 |
|  |  |  |  | FVRS | 0.02 | 0.001 |  |
| **Connection: Salience; Anterior cingulate cortex – SMN; Superior** | | | | | | | |
| Age-group | 0.15 | 0.001 | 0.17 | Age-group | 0.11 | 0.012 | 0.25 |
|  |  |  |  | FVRS | 0.01 | 0.017 |  |
| **Connection: Salience; Anterior cingulate cortex – Salience; Anterior insula R** | | | | | | | |
| Age-group | -0.14 | 0.003 | 0.14 | Age-group | -0.10 | 0.030 | 0.21 |
|  |  |  |  | FVRS | -0.01 | 0.025 |  |
| **Connection: Salience; Anterior insula R – Visual; Lateral R** | | | | | | | |
| Age-group | 0.11 | 0.006 | 0.12 | Age-group | 0.08 | 0.045 | 0.19 |
|  |  |  |  | FVRS | 0.01 | 0.040 |  |
| **Connection: Salience; Supramarginal gyrus R – Visual medial** | | | | | | | |
| Age-group | 0.12 | 0.007 | 0.12 | Age-group | 0.08 | 0.062 | 0.22 |
|  |  |  |  | FVRS | 0.01 | 0.011 |  |
| **Connection: DMN; Lateral parietal R – Visual medial** | | | | | | | |
| Age-group | 0.12 | 0.004 | 0.13 | Age-group | 0.09 | 0.037 | 0.21 |
|  |  |  |  | FVRS | 0.01 | 0.020 |  |
| **Connection: DMN; Lateral parietal L – Salience; Supramarginal gyrus R** | | | | | | | |
| Age-group | 0.14 | 0.003 | 0.14 | Age-group | 0.10 | 0.034 | 0.22 |
|  |  |  |  | FVRS | 0.01 | 0.015 |  |
| **Connection: DMN; Posterior parietal cortex – DAN; Intraparietal sulcus L** | | | | | | | |
| Age-group | 0.14 | 0.006 | 0.12 | Age-group | 0.11 | 0.044 | 0.18 |
|  |  |  |  | FVRS | 0.01 | 0.044 |  |

In the models, young adults were coded 1 and older adults 2. DAN=dorsal attention network; DMN=default mode network; FPN=frontoparietal network; FRS=modified Framingham risk score; L=left hemisphere; R=right hemisphere; SMN=sensorimotor network

| **Table S4B: Fazekas’ score** | | | | | | | |
| --- | --- | --- | --- | --- | --- | --- | --- |
| **Connection: Salience; Supramarginal gyrus R – Salience; Supramarginal gyrus L** | | | | | | | |
| Model 1: Age-group alone | | | | Model 2: Age-group + Fazekas’ score | | | |
|  | β | p-value | r^2^ |  | β | p-value | r^2^ |
| Age-group | -0.23 | 0.001 | 0.17 | Age-group | -0.11 | 0.184 | 0.25 |
|  |  |  |  | Fazekas’ | -0.16 | 0.023 |  |
| **Connection: DMN; Posterior cingulate cortex – SMN; Superior** | | | | | | | |
| Age-group | 0.25 | <0.001 | 0.28 | Age-group | 0.15 | 0.021 | 0.34 |
|  |  |  |  | Fazekas’ | 0.12 | 0.023 |  |

In the models, young adults were coded 1 and older adults 2. DMN=default mode network; L=left hemisphere; R=right hemisphere; SMN=sensorimotor network

| **Table S4C: White matter volume (WMV) cm^3^** | | | | | | | |
| --- | --- | --- | --- | --- | --- | --- | --- |
| **Connection: FPN; Posterior parietal cortex R – FPN; Lateral prefrontal cortex L** | | | | | | | |
| Model 1: Age-group alone | | | | Model 2: Age-group + WMV | | | |
|  | β | p-value | r^2^ |  | β | p-value | r^2^ |
| Age-group | -0.16 | 0.002 | 0.15 | Age-group | -0.12 | 0.015 | 0.27 |
|  |  |  |  | WMV | 0.001 | 0.003 |  |
| **Connection: FPN; Posterior parietal cortex R – DAN; Intraparietal sulcus R** | | | | | | | |
| Age-group | 0.19 | 0.001 | 0.17 | Age-group | 0.16 | 0.006 | 0.24 |
|  |  |  |  | WMV | -0.001 | 0.027 |  |
| **Connection: FPN; Posterior parietal cortex L – DAN; Intraparietal sulcus L** | | | | | | | |
| Age-group | 0.14 | 0.003 | 0.14 | Age-group | 0.11 | 0.018 | 0.24 |
|  |  |  |  | WMV | -0.001 | 0.007 |  |
| **Connection: DAN; Intraparietal sulcus R – SMN; Lateral L** | | | | | | | |
| Age-group | -0.14 | 0.003 | 0.14 | Age-group | -0.12 | 0.013 | 0.21 |
|  |  |  |  | WMV | 0.001 | 0.040 |  |
| **Connection: Salience; Anterior cingulate cortex – SMN; Lateral L** | | | | | | | |
| Age-group | 0.16 | <0.001 | 0.20 | Age-group | 0.18 | <0.001 | 0.27 |
|  |  |  |  | WMV | 0.001 | 0.025 |  |
| **Connection: Salience; Anterior cingulate cortex – SMN; Lateral R** | | | | | | | |
| Age-group | 0.13 | 0.006 | 0.12 | Age-group | 0.15 | 0.001 | 0.21 |
|  |  |  |  | WMV | 0.001 | 0.017 |  |
| **Connection: Salience; Anterior cingulate cortex – SMN; Superior** | | | | | | | |
| Age-group | 0.15 | 0.001 | 0.17 | Age-group | 0.18 | <0.001 | 0.27 |
|  |  |  |  | WMV | 0.001 | 0.006 |  |
| **Connection: Salience; Anterior insula L – SMN; Lateral L** | | | | | | | |
| Age-group | 0.16 | 0.001 | 0.18 | Age-group | 0.19 | <0.001 | 0.24 |
|  |  |  |  | WMV | 0.001 | 0.033 |  |
| **Connection: Salience; Rostral prefrontal cortex L – SMN; Superior** | | | | | | | |
| Age-group | 0.14 | <0.001 | 0.19 | Age-group | 0.17 | <0.001 | 0.27 |
|  |  |  |  | WMV | 0.001 | 0.020 |  |
| **Connection: Salience; Rostral prefrontal cortex R – Salience; Anterior insula R** | | | | | | | |
| Age-group | -0.21 | <0.001 | 0.25 | Age-group | -0.18 | <0.001 | 0.31 |
|  |  |  |  | WMV | 0.001 | 0.037 |  |
| **Connection: Salience; Anterior cingulate cortex – DAN; Intraparietal sulcus L** | | | | | | | |
| Age-group | 0.12 | 0.006 | 0.12 | Age-group | 0.15 | 0.001 | 0.20 |
|  |  |  |  | WMV | 0.001 | 0.023 |  |
| **Connection: Salience; Anterior insula R – Visual; Lateral R** | | | | | | | |
| Age-group | 0.11 | 0.006 | 0.12 | Age-group | 0.13 | 0.001 | 0.19 |
|  |  |  |  | WMV | 0.001 | 0.037 |  |
| **Connection: DMN; Lateral parietal L – Salience; Rostral prefrontal cortex R** | | | | | | | |
| Age-group | 0.14 | 0.001 | 0.17 | Age-group | 0.12 | 0.007 | 0.24 |
|  |  |  |  | WMV | -0.001 | 0.025 |  |
| **Connection: DMN; Posterior cingulate cortex – Salience; Supramarginal gyrus R** | | | | | | | |
| Age-group | 0.20 | <0.001 | 0.21 | Age-group | 0.17 | 0.002 | 0.29 |
|  |  |  |  | WMV | -0.001 | 0.011 |  |
| **Connection: DMN; Medial prefrontal cortex – Visual; Lateral L** | | | | | | | |
| Age-group | -0.10 | 0.007 | 0.12 | Age-group | -0.13 | 0.001 | 0.22 |
|  |  |  |  | WMV | -0.001 | 0.008 |  |

In the models, young adults were coded 1 and older adults 2. DAN=dorsal attention network; DMN=default mode network; FPN=frontoparietal network; L=left hemisphere; R=right hemisphere; SMN=sensorimotor network; WMV=white matter volume
